# Natural polysaccharides targeting mitochondrial function for colorectal cancer prevention and treatment: mechanisms and nano-delivery strategies

**DOI:** 10.1186/s13020-026-01463-4

**Published:** 2026-07-14

**Authors:** Huan Yan, Lianfeng Wang, Jiaxin Gan, Xin Zhou, Yuxin Jiang, Honglian Wang, Yuting Luo, Juan Fu, Jianqin Liu, Zhi Li

**Affiliations:** 1 College of Integrated Traditional Chinese and Western Medicine, Southwest Medical University, No. 1, Section 1, Xianglin Road, District of Longmatan, Luzhou, Sichuan Province 646000 China; 2 School of Integrated Traditional Chinese and Western Clinical Medicine, North Sichuan Medical College, No. 1, Yida North Road, District of Shunqing, Nanchong, Sichuan Province 637000 China; 3 The Key Laboratory of Integrated Traditional Chinese and Western Medicine for Prevention and Treatment of Digestive System Diseases of Luzhou City, The Affiliated Traditional Chinese Medicine Hospital, Southwest Medical University, No. 182, Chunhui Road, District of Longmatan, Luzhou, Sichuan Province 646000 China

**Keywords:** Natural polysaccharides, Colorectal cancer, Mitochondrial dysfunction, Nano-delivery systems, Oxidative stress, Chemotherapy resistance

## Abstract

**Background:**

Colorectal cancer (CRC) is a malignant tumor of the digestive system with high incidence and mortality worldwide. Mitochondrial dysfunction plays a central role in the development, progression, and chemotherapy resistance of CRC. Natural polysaccharides have attracted increasing attention in anti-CRC research in recent years due to their wide sources, low toxicity, good biocompatibility, and multi-target activities.

**Objective:**

To systematically review the molecular mechanisms by which natural polysaccharides target mitochondrial dysfunction to prevent and treat CRC, and to summarize the research progress of nano-delivery strategies.

**Methods:**

A systematic search was conducted in PubMed, Web of Science, Scopus, and SinoMed databases for literature published from January 1, 2015 to June 3, 2026. A total of 89 articles were included for analysis.

**Results:**

Natural polysaccharides exert anti-CRC effects through synergistic actions of multiple pathways, including regulation of reactive oxygen species homeostasis, induction of mitochondrial apoptosis and autophagy, remodeling of the tumor immune microenvironment, and protection of intestinal barrier function. Their bioactivities are closely related to molecular weight, monosaccharide composition, glycosidic bond type, branching structure, and chemical modification. Nano-delivery technologies can significantly improve their targeting efficiency and therapeutic activity. Regarding clinical studies, current evidence mainly focuses on the combined use of natural polysaccharides with chemotherapy and the management of cancer-related symptoms; direct clinical evidence targeting CRC resistance remains limited.

**Conclusion:**

Mitochondria-targeting nano-systems based on natural polysaccharides provide a promising multi-target strategy for CRC prevention, treatment, and reversal of chemotherapy resistance. However, current evidence has limitations, including inconsistent structural characterization, lack of direct co-localization evidence, and insufficient pharmacokinetic-pharmacodynamic evaluations.

## Introduction

Gastrointestinal malignancies account for nearly one-third of global cancer deaths, representing 5.26 million new cases and 3.7 million deaths in 2021, with colorectal cancer (CRC) exhibiting the highest incidence and mortality rates [1]. CRC is the third most common cancer in men and the second in women [2]. Its global burden is projected to rise annually, reaching 3.2 million new cases and 1.6 million deaths by 2040 [3]; by 2050, annual deaths are expected to hit 2.18 million, with disability-adjusted life years (DALYs) climbing to 41.7 million [4]. CRC development is a protracted process influenced by complex interactions among genetic and environmental factors, including sedentary lifestyles, obesity, alcohol consumption, smoking, and gut microbiota [5]. Current prevention and treatment strategies primarily rely on surgery, radiotherapy, chemotherapy, and immunotherapy; however, challenges such as postoperative complications, drug resistance, and toxic side effects remain unresolved.

Mitochondria, as the cellular powerhouses, play a pivotal regulatory role in tumour metabolic reprogramming, proliferation, survival, and metastasis [6]. Mitochondrial dysfunction refers to abnormalities in energy production, metabolic regulation, or cellular signalling, ultimately leading to intrinsic apoptosis. This dysfunction modulates intestinal inflammation through mechanisms involving mitochondrial injury, mitochondrial DNA (mtDNA) release, mitochondrial oxidative stress, and mitophagy [7]. Research indicates that chronic intestinal inflammation induces mitochondrial metabolic reprogramming and dysfunction, thereby promoting the malignant progression of inflammatory bowel disease and the development of drug-resistant phenotypes in CRC [8, 9].

Natural polysaccharides constitute a class of low-toxicity, biocompatible macromolecular compounds formed by the polymerization of monosaccharides via glycosidic bonds, which are widely occurring in plants, animals, and microorganisms [10]. These compounds exhibit multiple biological activities, including antioxidant, anti-inflammatory, immunomodulatory, and anticancer effects [11]. They improve CRC outcomes by regulating gut microbiota [12], counteracting oxidative stress [13], mitigating mitochondrial dysfunction [14], and protecting the intestinal mucosal barrier [15]. Notably, they hold promise as adjunctive agents that complement surgery, radiotherapy, chemotherapy, and immunotherapy by targeting mitochondrial metabolic reprogramming, inducing tumour cell apoptosis, and modulating anti-tumour immunity.

Current research on natural polysaccharides against CRC is primarily preclinical, with limited clinical applications or observational studies. This may be attributed to their relatively low bioavailability. Nanodelivery technologies, however, enable targeted delivery of these compounds to tumour tissues. Commonly employed nanocarriers include silver nanoparticles, selenium nanoparticles, poly(lactic-co-glycolic acid) (PLGA) nanoparticles, polyethylene glycol (PEG)-modified nanoparticles, and nanoemulsions. Existing evidence indicates that the combination of natural polysaccharides with nanotechnology exhibits promising synergistic antitumour activity both in vitro and in vivo, as demonstrated with polysaccharides from *Lentinula edodes* [16], *Astragalus membranaceus* [17], *Cordyceps sinensis* [18], and *Laminaria japonica* [19]. This approach holds promise for enhancing the bioavailability and clinical application of natural polysaccharides.

Research on nano-delivery technology in the treatment of CRC with natural polysaccharides has made some progress, but current studies remain relatively fragmented and few clinical trials are available. Systematic reviews that integrate natural polysaccharides with mitochondrial targeting are still lacking. Therefore, this review systematically dissects the molecular mechanisms by which natural polysaccharides target mitochondrial dysfunction to prevent and treat CRC, with an emphasis on the structure–activity relationship and the synergistic effects of nano-delivery technology. This aims to provide a reference for developing natural polysaccharide-based anti-CRC strategies that reverse chemotherapy resistance.

## Materials and methods

A systematic literature search was conducted for articles published between January 1, 2015 and June 3, 2026, aiming to comprehensively review the application and research progress of natural polysaccharides targeting mitochondria via nano-delivery systems in the treatment of CRC. The following electronic databases were searched: PubMed, Web of Science, Scopus, and SinoMed. The search strategy combined keywords and Medical Subject Headings (MeSH) around four core dimensions: (1) therapeutic target-colorectal cancer; (2) active component-natural polysaccharides; (3) mechanism target-mitochondria; and (4) delivery strategy-nano-delivery systems. Boolean operators “AND” and “OR” were used to combine search terms, ensuring both comprehensiveness and accuracy.

The core search terms were as follows: (1) CRC-related: colorectal cancer, CRC, colorectal neoplasms, colon carcinoma; (2) Natural polysaccharide-related: natural polysaccharides, plant polysaccharides, bioactive polysaccharides; (3) Mitochondria-related: mitochondria, mitochondrial dysfunction, mitochondrial apoptosis, mitophagy, oxidative phosphorylation; (4) Nano-delivery-related: nanoparticles, nanocarriers, nanodelivery, nanoformulations, drug delivery systems, targeted delivery, mitochondria-targeted.

Inclusion criteria: (1) Study type: original research articles investigating the effects of natural polysaccharides on CRC cells or animal models; (2) Mechanistic studies: studies elucidating the molecular mechanisms by which natural polysaccharides exert anti-CRC effects through mitochondrial targeting; (3) Delivery studies: studies involving nano-delivery systems loaded with natural polysaccharides for CRC treatment. Additionally, all included animal studies were required to be reported in accordance with the ARRIVE guidelines [20, 21].

Exclusion criteria: (1) Non-English articles; (2) Non-original studies such as reviews, conference abstracts, commentaries, and case reports; (3) Studies involving natural polysaccharides but not CRC; (4) Studies involving CRC but where natural polysaccharides were not the primary intervention; (5) Studies using compound formulations from which the independent effect of natural polysaccharides could not be isolated.

The following key information was extracted from the finally included articles: study model (cell lines, animal models), type and source of natural polysaccharide, type of nano-delivery system (material, particle size, modification method), administration route and dosage, mitochondria-related parameters (MMP (mitochondrial membrane potential), ROS (reactive oxygen species) levels, ATP (adenosine triphosphate) production, apoptosis-related proteins, autophagy-related proteins), anti-tumor effects (inhibition of cell proliferation, induction of apoptosis, cell cycle arrest, in vivo tumor suppression), and changes in major signaling pathways, such as *AMPK/mTOR* (AMP-activated protein kinase/mechanistic target of rapamycin), *PINK1/Parkin* (PTEN*-*induced putative kinase 1/*PARK2* (E3 ubiquitin ligase)) and Nrf2 (nuclear factor erythroid 2-related factor 2).

**Fig. 1:**
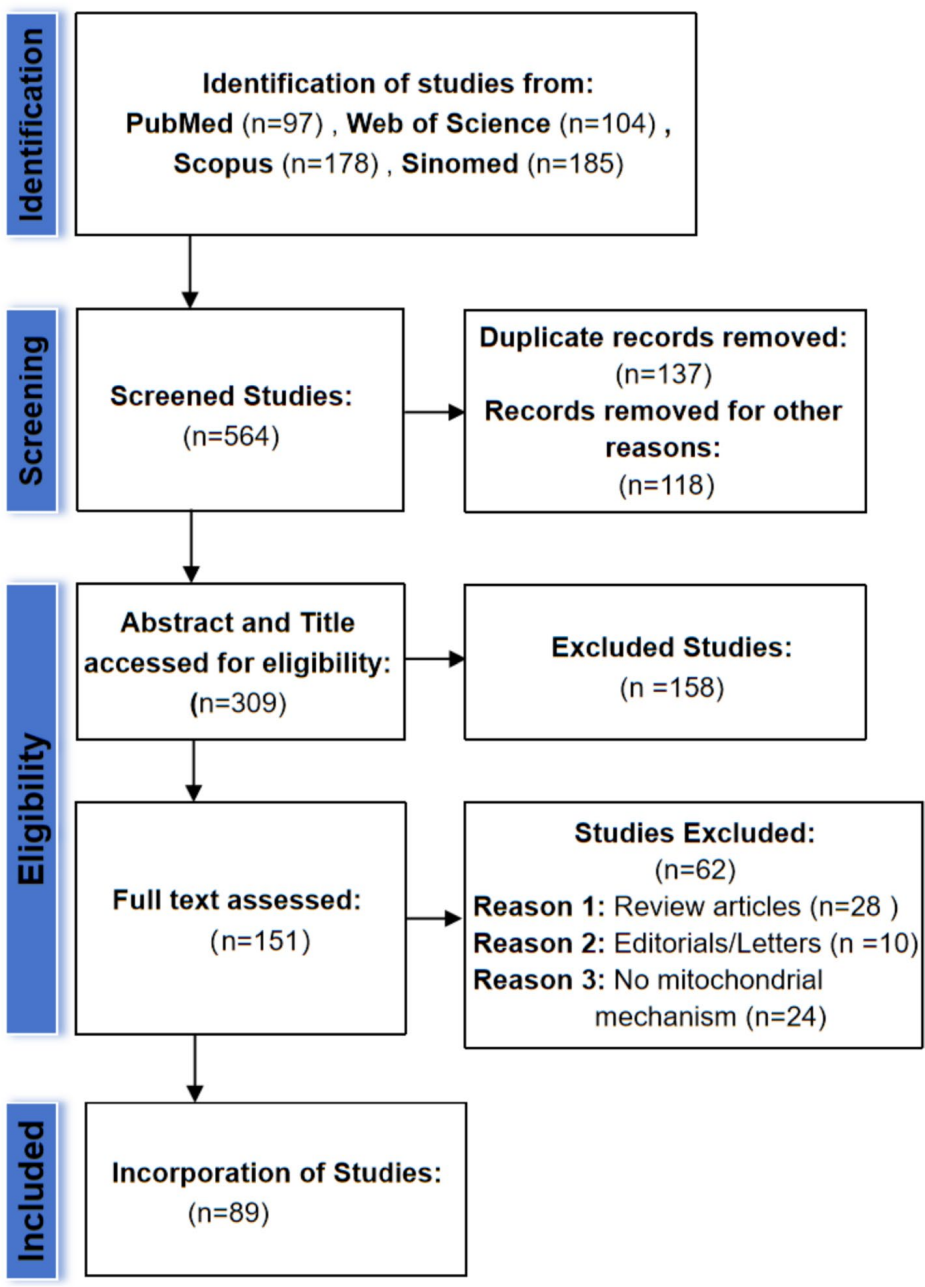
Identification of studies via databases

The literature screening process followed the PRISMA (Preferred Reporting Items for Systematic Reviews and Meta-Analyses) guidelines. A total of 564 records were initially retrieved. After removing duplicate records and screening by titles and abstracts, 151 articles remained for full-text assessment. Following full-text review, 89 articles were finally included in the systematic analysis. Literature screening and data extraction were carried out independently by two researchers and then cross-validated. Any disagreements were settled through discussion or by referral to a third researcher. The detailed screening process, including reasons for exclusion at each stage, is illustrated in Fig. 1.

## Relationship between the structure of natural polysaccharides and their anti-CRC activity

**Fig. 2:**
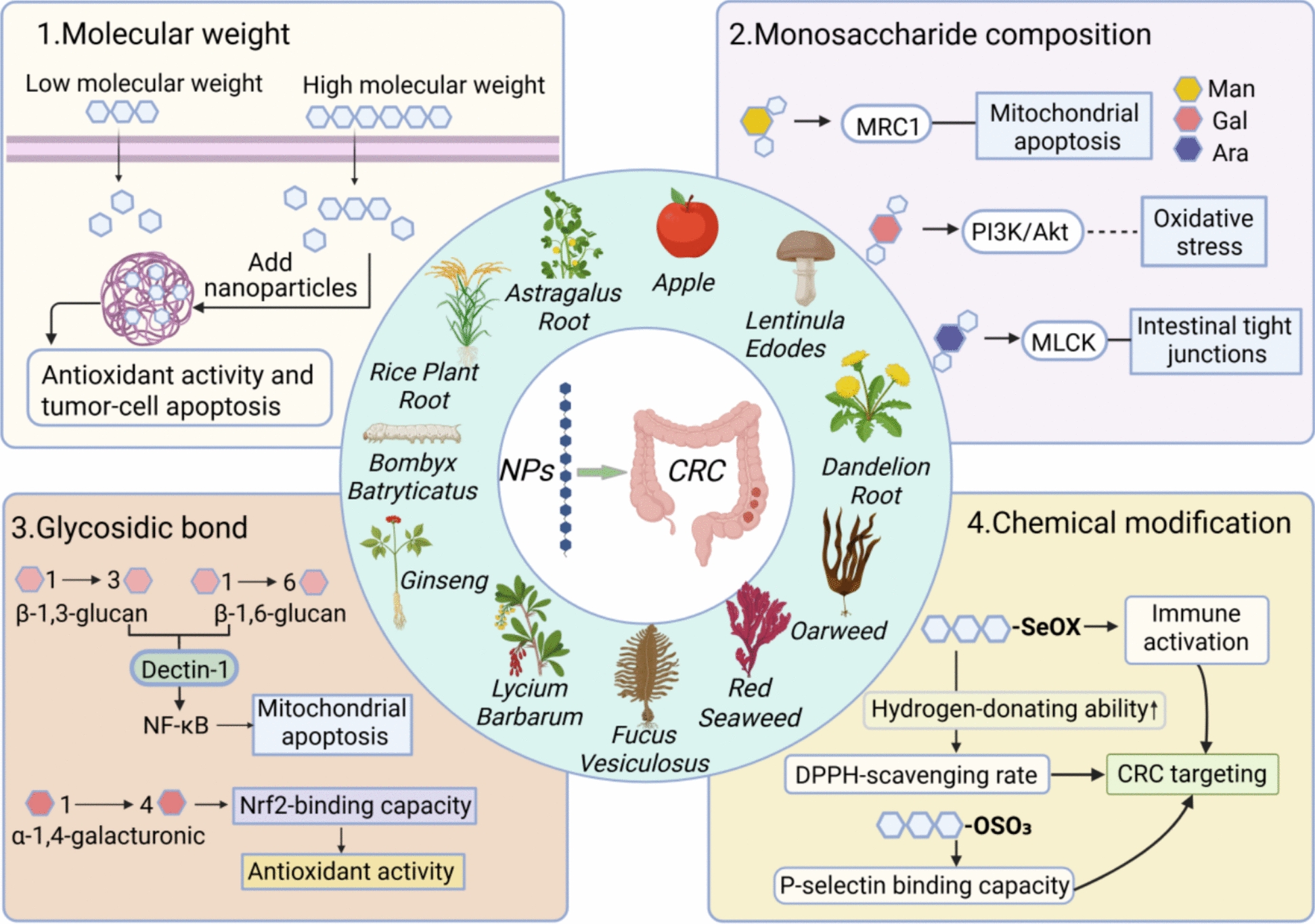
Relationship between the structural characteristics of natural polysaccharides and their anti-CRC properties. Low-molecular-weight polysaccharides readily penetrate cell membranes and induce mitochondrial apoptosis, whereas high-molecular-weight polysaccharides can have their bioavailability improved through nano-delivery. Monosaccharide composition (e.g., mannose, Man; galactose, Gal; arabinose, Ara) determines the binding of polysaccharides to pattern recognition receptors (PRRs), thereby regulating mitochondrial apoptosis, oxidative stress, and intestinal tight junctions. The type of glycosidic bond (e.g., β−1,3-glucan, β−1,6-glucan) affects Dectin-1 receptor recognition and the NF-κB (Nuclear factor kappa-light-chain-enhancer of activated B cells) signaling pathway, as well as nuclear factor erythroid 2-related factor 2 (*Nrf2*) binding capacity and antioxidant activity. Chemical modifications (e.g., selenylation, sulfation) enhance antioxidant activity and P-selectin binding ability, thereby improving targeting efficiency against CRC. Arrows in the figure indicate regulatory relationships between structural parameters and functions. Detailed mechanisms and supporting literature are presented in Sect. "Molecular weight regulates bioavailability and mode of action"–"Chemical modification remodels physicochemical properties and anti-tumor function"

Natural polysaccharides are a class of high-molecular-weight polymers formed by the glycosidic linkage of monosaccharides. They are widely distributed among plants, microorganisms (including bacteria and fungi), and marine organisms, exhibiting marked variations in structural units, glycosidic linkage patterns, and physicochemical properties depending on their source. Based on origin, natural polysaccharides can be categorized into plant-derived polysaccharides, such as *Astragalus membranaceus* polysaccharide (APS), *Lycium barbarum* polysaccharide (LBP), and *Angelica sinensis* polysaccharide (AS); fungal-derived polysaccharides, including lentinan (LNT), *Ganoderma lucidum* polysaccharide (GLP), and *Cordyceps sinensis* polysaccharide; microbial polysaccharides, such as lactic acid bacterial exopolysaccharides; and seaweed polysaccharides, such as fucoidan (FU) and alginate [10, 22]. From a chemical structural perspective, polysaccharides are divided into homopolysaccharides composed of a single type of monosaccharide, e.g., starch and cellulose, and heteropolysaccharides composed of two or more types of monosaccharides, e.g., pectin and hemicellulose. In terms of physiological function, natural polysaccharides can be classified as storage polysaccharides (e.g., starch and glycogen), structural polysaccharides (e.g., cellulose and chitin), and gel or mucilaginous polysaccharides (e.g., hyaluronic acid (HA) and pectin) [23]. Furthermore, depending on the presence of functional groups such as sulfate, acetyl, or uronic acid moieties, polysaccharides can be further divided into neutral polysaccharides, acidic polysaccharides, and sulfated polysaccharides. Notably, sulfated polysaccharides generally exhibit superior anti-inflammatory and anti-tumor activities compared with their neutral counterparts [22]. Of particular interest, exopolysaccharides derived from probiotics represent an important subset of microbial polysaccharides and have demonstrated anti-tumor potential by modulating intestinal microecology, inducing apoptosis in CRC cells, and enhancing immune responses [24]. A clear classification of natural polysaccharides facilitates the understanding of their structure–activity relationships [25]. We found that four aspects—molecular weight, monosaccharide composition, glycosidic bond and branching structure, and chemical modification**—**are closely related to the biological activities of natural polysaccharides (Fig. 2). This section also systematically discusses the potential links between these parameters, along with extraction methods, and anti-CRC activity.

### Molecular weight regulates bioavailability and mode of action

Molecular weight is a key factor influencing the bioavailability and action mode of natural polysaccharides. Generally, low-molecular-weight polysaccharides possess better solubility and cellular permeability, making them more capable of entering tumor cells and directly acting on subcellular structures such as mitochondria. For example, SVP-A-1, a polysaccharide from *Sanghuangporus vaninii* with a molecular weight of 22.5 kDa, can easily penetrate cell membranes due to its small size and induce mitochondrial oxidative damage and apoptosis through ROS accumulation [26]. In contrast, high-molecular-weight polysaccharides, although often exhibiting more complex spatial conformations and more potential binding sites, have relatively limited penetration and transmembrane transport capacity in tumor tissues. DOP, a polysaccharide from *Dendrobium officinale* with a molecular weight of approximately 730 kDa, can induce autophagy via the ROS-AMPK-mTOR pathway, but its high molecular weight may restrict its diffusion efficiency within the tumor microenvironment [14]. Nevertheless, the limitations of high molecular weight can be overcome by designing appropriate delivery systems. For instance, after *Cordyceps sinensis* extracellular polysaccharides (EPS) was formulated with selenium nanoparticles into a 80–125 nm composite system, its antioxidant capacity and pro-apoptotic targeting against tumor cells were significantly enhanced [18, 27]. Therefore, molecular weight not only affects the potency of bioactivity but also determines, to some extent, whether a polysaccharide tends to exert direct intracellular intervention or relies on delivery and immunomodulation to exert its anti-CRC effects.

### Monosaccharide composition affects receptor recognition and functional bias

Natural polysaccharides are often composed of monosaccharides such as xylose, glucose, rhamnose, mannose, fucose, galactose, and arabinose [28]. Variations in their molar ratios can significantly alter the interactions between polysaccharides and receptors or signaling pathways. Studies have shown that mannose is readily recognized by PRRs on the surface of macrophages, B cells, and dendritic cells (DCs) [29, 30]; therefore, mannose-rich polysaccharides generally possess strong immune-targeting potential. ABPA1 (*Aloe vera* acetylated mannan) is a typical mannose-dominated acetylated mannan with a molecular weight of approximately 360 kDa, consisting of 89.5% mannose, 6.1% glucose, 3.6% galactose, and 0.8% arabinose [31]. ABPA1 can target macrophages via mannose receptors such as *MRC1* (Mannose Receptor C-Type 1) and activate the mitochondrial apoptosis pathway in CRC cells. Galactose/glucose-rich polysaccharides are more likely to participate in the alleviation of oxidative stress and maintenance of mitochondrial homeostasis. For example, SCP3 (sulfated *Cyclocarya paliurus* polysaccharide 3) has a galactose content of 42.9% and may reduce oxidative damage through *PI3K/AKT* (Phosphoinositide 3-kinase/Protein Kinase B signaling pathway)-related signaling [32]. Fucose-rich polysaccharides also exhibit unique advantages. For instance, ACP (*Agrocybe cylindracea* fucoglucogalactan) contains 13.2% fucose and can upregulate *CTSD* (cathepsin D) via H3K27ac (histone H3 lysine 27 acetylation) epigenetic modification, thereby inducing lysosome-mitochondria crosstalk-mediated apoptosis [33]. Arabinose-related components may improve intestinal barrier function by inhibiting the *MLCK* (myosin light chain kinase) pathway, thus indirectly optimizing the intestinal microenvironment and mitochondrial function [34]. Therefore, monosaccharide composition primarily determines the receptor recognition preference and functional orientation of natural polysaccharides, serving as an important basis for their immunomodulatory, metabolic intervention, and barrier-protective effects.

### Glycosidic bond and branching structure influence recognition mode and membrane interaction

The configuration and linkage type of glycosidic bonds determine the flexibility, spatial arrangement, and receptor recognition ability of polysaccharide chains, making them key factors affecting receptor binding mode and membrane interaction capacity. In general, β-configured polysaccharides, particularly β−1,3/1,6-glucans, are more readily recognized by PRRs such as Dectin-1, and therefore exhibit higher activity in immunomodulation and inflammation control. For example, LMW-AP-FBG, a low-molecular-weight β−1,3–1,6-glucan derived from *Aureobasidium pullulans*, can activate NF-κB-related signaling via the Dectin-1 receptor, downregulate pro-inflammatory cytokines including TNF-α (tumor necrosis factor alpha), IL-6 (interleukin-6), and IL-1β, modulate apoptosis-related proteins such as Bcl-2 (B-cell lymphoma 2) and Bcl-xL (B-cell lymphoma-extra large), and ultimately induce mitochondria-mediated apoptosis [35]. In contrast, a pectic polysaccharide isolated from *Chuanminshen violaceum* (CVP-AP-I), possesses a linear backbone of α−1,4-galacturonic acid, which is relatively regular and may facilitate interaction with the *Nrf2*-associated antioxidant system, thereby enhancing cellular antioxidant capacity [36]. Branching structure further influences the higher-order conformation and exposure of functional sites of polysaccharides. Highly branched polysaccharides often have more complex spatial morphology and richer binding interfaces, which may enhance their interaction with cell or subcellular membranes. For instance, the mannose residues of ABPA1 are randomly acetylated at the O-2, O-3, and O-6 positions. This structural feature, characterized by branching accompanied by increased hydrophobicity, facilitates its binding to mitochondrial membranes and induces cytochrome c release [31]. Therefore, while monosaccharide composition determines the receptor recognition tendency of polysaccharides, glycosidic bond and branching structure affect their binding mode and mechanism of action. Together, these two aspects constitute an important structural basis for the biological effects of natural polysaccharides.

### Chemical modification remodels physicochemical properties and anti-tumor function

Chemical modification represents an important strategy for regulating the activity of natural polysaccharides during structural optimization. The introduction of functional groups such as sulfate or selenide moieties can alter the charge distribution, redox properties, and intermolecular interactions of polysaccharides. Sulfation generally enhances the binding ability of polysaccharides to molecules such as P-selectin, thereby improving their targeted delivery potential in the CRC cell or tumor vascular microenvironment [37]. Taking DP, dandelion root polysaccharide, as an example, the unmodified DP exerts protective effects in a mouse model of ulcerative colitis by activating the *Nrf2/HO-1/NQO-1* (nuclear factor erythroid 2-related factor 2/heme oxygenase-1/NADH quinone oxidoreductase 1) pathway, inhibiting ferroptosis in intestinal epithelial cells (IECs), repairing the intestinal barrier, and modulating gut microbiota metabolism [38]. Chemical modification can further alter the physicochemical properties and functions of DP. After selenylation, the molecular weight of DP decreases and its hydrogen-donating capacity increases, resulting in a DPPH (2,2-diphenyl-1-picrylhydrazyl) radical scavenging rate approximately two times higher than that of the unmodified polysaccharide. In addition, selenylated DP promotes macrophage proliferation and phagocytosis [39]. Previous studies have shown that the antioxidant activity of polysaccharides is closely related to their hydrogen-donating capacity [40], and the introduction of selenium atoms may reduce the dissociation energy of relevant hydrogen bonds. After sulfation, DP adopts a triple-helix conformation with the introduction of sulfate groups, exhibiting enhanced antioxidant capacity and Fe^2^⁺ chelating ability, as well as inhibitory effects on α-amylase and α-glucosidase and the ability to promote probiotic proliferation [41]. In summary, chemical modification is not a simple addition to the native activity of natural polysaccharides, but rather a directed functional adjustment achieved by altering key parameters such as molecular weight, functional groups, and spatial conformation. Although not all of the above-mentioned chemical modification studies were directly conducted using CRC models, the structure–activity relationships they reveal may provide useful references for designing polysaccharide modification strategies targeting CRC.

### Extraction and preparation processes affect structural preservation and activity expression

Although extraction and preparation processes are not intrinsic structural parameters of natural polysaccharides, they significantly influence molecular weight distribution, branch retention, stability of active groups, and final purity, thereby affecting structural characterization results and biological effects. Therefore, when analyzing the structure–activity relationship of natural polysaccharides for CRC prevention and treatment, extraction and preparation processes are not merely pretreatment steps but rather critical factors that influence activity expression. This is particularly true for polysaccharides with high structural heterogeneity, where different extraction conditions may lead to variations in solubility, conformational stability, and receptor recognition ability of the obtained fractions, consequently affecting their antioxidant, barrier-protective, and anti-tumor activities. In recent years, various green and enhanced extraction techniques have been developed to improve extraction efficiency and reduce structural damage caused by prolonged high-temperature treatment. For example, ultrasound-assisted extraction (UAE), has been shown to increase the yield of APS and improve their antioxidant activity [42]. Deep eutectic solvent (DES), combined with ultrasound or enzyme-assisted extraction of polysaccharides from *Astragalus membranaceus* and *Gastrodia elata* not only enhances the yield but also reduces molecular weight while preserving monosaccharide composition and bioactivity [43, 44]. Polysaccharides extracted from *Panax quinquefolius* using a DES system also exhibit good activity in intestinal epithelial protection and intestinal barrier maintenance [45]. DES-enhanced ultrasound extraction of LNT similarly demonstrates superior antioxidant capacity [46]. In addition, microwave puffing pretreatment can increase the extraction yield of *Dendrobium officinale* polysaccharides and reduce structural damage caused by heat treatment to some extent [47].

In summary, the anti-CRC activity of natural polysaccharides is not determined by a single factor, but rather results from the combined effects of molecular weight, monosaccharide composition, glycosidic linkage and branching structure, chemical modification, and extraction/preparation processes. Therefore, when subsequently discussing the mechanisms by which natural polysaccharides participate in CRC prevention and treatment through the regulation of oxidative stress, mitochondrial apoptosis and autophagy, immune metabolism, and intestinal homeostasis, these structural bases should be taken into consideration.

## Natural polysaccharides prevent and treat CRC by modulating mitochondrial function

**Fig. 3:**
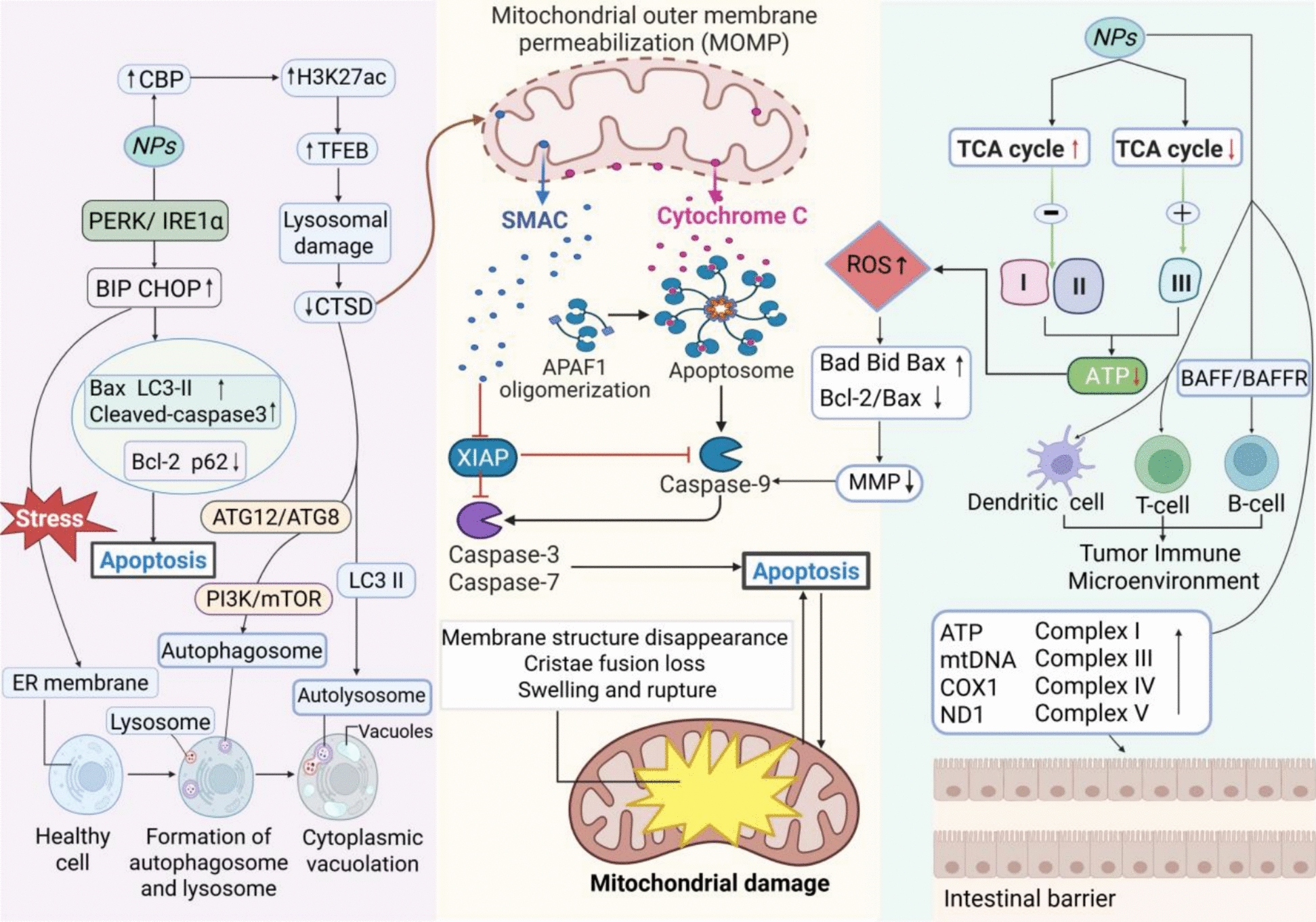
Schematic diagram of the mechanism by which natural polysaccharides modulate mitochondrial function to combat CRC. Natural polysaccharides trigger apoptosis by inducing mitochondrial ROS accumulation and loss of MMP. This process involves the regulation of *Bax* (*2*-associated X protein)/*Bcl-2* and *caspase-3/9* (cysteine-aspartic acid protease 3/9). These polysaccharides also modulate mitophagy mediated by *PINK1*/*Parkin* and *BNIP3* (*BCL2* interacting protein 3). In addition, they improve mitochondrial metabolism in immune cells, thereby enhancing anti-tumor immunity. They also protect mitochondrial respiratory chain complexes and mtDNA-encoded genes in IECs, which helps maintain the intestinal barrier. Furthermore, natural polysaccharides influence mitochondria-endoplasmic reticulum interactions involving *BIP*/*CHOP* (binding immunoglobulin protein/CCAAT/enhancer-binding protein homologous protein) and lysosome-mitochondria crosstalk mediated by *CTSD*. Detailed mechanisms are described in Sect. "Natural polysaccharides prevent and treat CRC by modulating mitochondrial function" and Table 2

Mitochondria serve as a central hub for cellular energy metabolism, redox homeostasis, and signal transduction. Their functional integrity is essential for maintaining intestinal epithelial integrity, immune balance, and the regulation of cell fate. During the development and progression of CRC, mitochondrial dysfunction is extensively involved in pathological processes such as tumor cell proliferation, metastasis, drug resistance, and remodeling of the tumor microenvironment. Mitochondria-derived ROS are often considered a key mediator linking mitochondrial damage to downstream signaling abnormalities [48]. Recent studies have shown that natural polysaccharides can intervene in CRC-related mitochondrial dysfunction by regulating mitochondrial oxidative stress, apoptosis, autophagy, immune metabolism, and intestinal barrier homeostasis. Their effects are also closely associated with functional crosstalk between mitochondria and other organelles (Fig. 3). Based on these findings, the following sections will further elaborate on the possible mechanisms by which natural polysaccharides participate in CRC prevention and treatment through mitochondrial regulation, focusing on four aspects: ROS-related mitochondrial regulation, immune cell mitochondrial function, intestinal epithelial mitochondrial homeostasis, and mitochondria-organelle interactions.

### Natural polysaccharides modulate ROS-mediated mitochondrial apoptosis, autophagy, and energy metabolism

Mitochondria are both the main source of ROS and a primary target of ROS attack [49]. Under physiological conditions, ROS participate in signal transduction. However, under oxidative stress, excessive ROS accumulation leads to decreased MMP, increased membrane permeability, impaired ATP synthesis, and mtDNA damage. These changes further trigger apoptosis, abnormal autophagy, and amplified inflammatory responses [50, 51]. During IBD (Inflammatory Bowel Disease)-related inflammation and CRC development, the ROS-mitochondria axis serves as a key mechanistic link connecting chronic inflammation, metabolic abnormalities, and tumor progression.

Existing studies indicate that the regulation of the ROS-mitochondria axis by natural polysaccharides exhibits a clear context-dependent pattern. In CRC cells, some natural polysaccharides disrupt the redox homeostasis threshold by promoting ROS accumulation. This leads to loss of MMP, cytochrome c release, and caspase cascade activation, thereby initiating the mitochondrial apoptosis program. For example, SVP-A-1 induces ROS accumulation, promotes the translocation of pro-apoptotic proteins such as *Bax*, *Bad* (BCL2-associated agonist of cell death), and *Bid* (BH3 interacting domain death agonist) to mitochondria, and downregulates anti-apoptotic proteins, ultimately triggering the mitochondrial apoptosis pathway [26]. Similarly, *Hericium erinaceus* polysaccharide activates the caspase-9-dependent apoptotic pathway by regulating *Bcl-2/Bax* expression and reducing MMP [52]. Another plant polysaccharide, ABPA1, causes more direct ultrastructural damage, including fusion and disappearance of mitochondrial cristae as well as swelling and rupture [31]. Furthermore, a polysaccharide derived from *Lactobacillus casei* SB27 upregulates *HINT2* (histidine triad nucleotide-binding protein 2) and promotes its interaction with *MCU* (mitochondrial calcium uniporter). This interaction induces mitochondrial calcium overload, leading to loss of membrane potential, opening of the mitochondrial permeability transition pore (mPTP), and activation of caspase-3/8/9, ultimately resulting in apoptosis. Although this study did not measure ROS, calcium overload and ROS are causally linked, making this *HINT2/MCU*-calcium overload-apoptosis axis complementary to the ROS-dependent pathways described above [53]. In addition to the aforementioned mechanisms that directly or indirectly target mitochondria, acetylated *Dendrobium huoshanense* polysaccharide (DHP) exhibits another distinct mechanism. Specifically, DHP reduces Fas receptor sialylation by inhibiting ST6 beta-galactoside alpha-2,6-sialyltransferase 1 (ST6Gal-I), thereby restoring Fas receptor sensitivity to Fas ligand (FasL) and activating the death receptor pathway. This subsequently links to mitochondria via truncated Bid (tBid), leading to a decline in mitochondrial membrane potential, an elevated Bax/Bcl-2 ratio, and activation of caspase-9 and caspase-3. Concurrently, DHP suppresses the PI3K/AKT pathway to decrease Uridine diphosphate N-acetylglucosamine (UDP-GlcNAc) levels, reverses metabolic reprogramming, and ultimately induces apoptosis in HCT116 colorectal cancer cells [54].

In contrast, under inflammatory or non-tumor injury conditions, natural polysaccharides often exhibit a protective role by maintaining mitochondrial homeostasis. For example, APS alleviates mitochondrial metabolic stress by enhancing mitochondrial metabolism, inhibiting glycolysis, and reducing lactate accumulation. It also mitigates oxidative damage through the *ALDH1A1* pathway and restores ATP production, thereby improving cellular energy supply [55]. Similarly, *Hericium erinaceus* polysaccharide shows comparable effects. It enhances superoxide dismutase (SOD) activity, reduces ROS levels, and increases MMP. These changes effectively improve mitochondrial function and alleviate inflammation [56]. However, the same polysaccharide may exert different effects in different cell types. For instance, ABPA1 has opposite effects on the tricarboxylic acid cycle and respiratory chain complexes in RKO versus SW480 CRC cells, yet both ultimately lead to reduced ATP levels, ROS accumulation, and apoptosis [31]. It is worth noting that mitophagy mediated by pathways such as *PINK1/Parkin* (PTEN-induced putative kinase 1/*PARK2*, a gene encoding an E3 ubiquitin ligase) and *BNIP3* is an important adaptive process for eliminating damaged mitochondria. However, in tumor cells, this process may also be exploited to maintain metabolic advantage and cell survival [47, 57–60].

Therefore, the impact of natural polysaccharides on ROS-related mitochondrial function is not limited to simply promoting apoptosis or exerting antioxidant effects. Instead, it represents a comprehensive regulation of mitophagy, energy metabolism, and cell fate determination. In summary, the regulation of the ROS-mitochondria axis by natural polysaccharides is not unidirectional but exhibits bidirectional characteristics depending on cell status and pathological context. This is one of the important mechanistic bases for their involvement in CRC prevention and treatment.

### Natural polysaccharides modulate mitochondrial function in immune cells to restore intestinal immune homeostasis

Mitochondria are not only the central hub of cellular energy metabolism but also an important platform for regulating immune cell activation, differentiation, and effector functions [61, 62]. In the tumor microenvironment (TME) of CRC, factors such as hypoxia, acidosis, and nutrient competition often lead to mitochondrial dysfunction in immune cells. This dysfunction results in mtDNA damage, excessive ROS production, decreased ATP synthesis, and impaired metabolic adaptation, thereby compromising anti-tumor immune function [63, 64]. In tumor-associated macrophages (TAMs), *Drp1* (dynamin-related protein 1) -mediated imbalance of mitochondrial dynamics weakens their phagocytic and antigen-presenting capacities [65, 66]. Among T cell subsets, mitochondrial defects promote CD8⁺ T cell exhaustion through excessive ROS production [67] and also increase the apoptosis rate of regulatory T cells due to their sensitivity to oxidative stress [68]. Furthermore, mitochondrial damage impairs germinal center formation and antibody production in B cells by downregulating *TFAM* (mitochondrial transcription factor A) expression [69]. Therefore, understanding the immunomodulatory effects of natural polysaccharides from the perspective of mitochondrial function will help to more accurately elucidate their anti-CRC mechanisms.

Unlike the direct effects of polysaccharides on tumor cells, the following content focuses on how natural polysaccharides indirectly exert anti‑tumor effects by improving mitochondrial homeostasis in immune cells. In the AOM/DSS-induced colorectal cancer model, *Ganoderma lucidum* polysaccharide (GLP) induces mitochondrial fission by upregulating Drp1 and downregulating Optic atrophy 1 (OPA1), which is accompanied by a decrease in mitochondrial membrane potential and an elevation of ROS levels. Consequently, this drives the repolarization of tumor-associated macrophages (TAMs) from the pro-tumor M2 phenotype toward the anti-tumor M1 phenotype. Notably, treatment with the Drp1 inhibitor Mdivi-1 abrogates this effect, demonstrating that mitochondrial dynamics serves as an upstream determinant of immune remodeling [70]. Previous studies have shown that DOPS1, a polysaccharide from *Dendrobium officinale*, increases the number of mitochondria in CD8⁺ T cells and promotes ATP production, thereby improving their metabolic fitness. It also reduces the expression of programmed cell death protein 1 (PD-1), which reverses T cell exhaustion and ultimately enhances anti‑tumor immune responses [71]. In B cells, APS exhibits a dual regulatory role. On one hand, it enhances mitochondrial metabolism, promoting the secretion of anti‑inflammatory cytokines such as IL-10 and TGF-β1 (transforming growth factor beta 1) while inhibiting the release of pro-inflammatory cytokines including IL-6 and TNF-α. On the other hand, APS stabilizes the memory B cell population by regulating the BAFF/BAFFR (B-cell activating factor/BAFF receptor) signaling pathway, thereby improving pathological changes such as intestinal inflammatory infiltration and crypt structure [55]. Natural polysaccharides also play a prominent role in reshaping the balance between innate immunity and T cell subsets. SVP‑A‑1 promotes T cell proliferation and dendritic cell activation by regulating L‑arginine metabolism, thereby remodeling the anti‑tumor immune landscape [26].

Distinct from general immunomodulation, the effect of natural polysaccharides on the CRC immune microenvironment is not an isolated event. Rather, it is fundamentally grounded in their ability to improve mitochondrial function in immune cells. This process can be summarized as a cascade: mitochondrial metabolic remodeling, followed by restoration of immune function, and ultimately enhanced anti-tumor effects. A study investigating the reversal of TAM phenotypes via GLP-mediated regulation of mitochondrial dynamics in macrophages provides direct evidence for this process [70]. Although direct evidence remains to be strengthened, current studies suggest that targeting mitochondrial function in immune cells represents a key dimension through which natural polysaccharides reshape immune homeostasis in CRC.

### Natural polysaccharides modulate intestinal epithelial mitochondrial function to repair the intestinal barrier

The intestinal barrier is an important physiological foundation for maintaining intestinal homeostasis and preventing the translocation of pathogens and harmful metabolites. Its integrity highly depends on the normal energy metabolism and survival of IECs. Mitochondria, as the energy hub of IECs, can directly impair tight junctions, mucus secretion, and epithelial renewal through energy depletion, oxidative stress, and apoptosis when mitochondrial dysfunction occurs [72]. Conversely, the release of inflammatory factors and dysbiosis of the gut microbiota triggered by barrier damage further aggravate mitochondrial injury in IECs, forming a vicious cycle [73]. Intestinal barrier damage is closely associated with persistent chronic inflammation, microbial translocation, and inflammation-to-cancer transition. Therefore, protecting IEC mitochondria is considered a key mechanism by which natural polysaccharides exert their preventive and therapeutic effects against CRC.

Existing studies have shown that natural polysaccharides can alleviate inflammation‑related injury by improving mitochondrial energy metabolism in IECs, reducing mitochondria‑associated apoptosis, and maintaining epithelial barrier integrity. For example, *Cyclocarya paliurus* polysaccharide (CPP) and its sulfated derivative SCP3 protect IEC‑6 cells from injury. They achieve this by restoring MMP and inhibiting the expression of oxidative damage‑related proteins [74]. MGN‑3 (modified rice bran arabinoxylan) exhibits more comprehensive repair capacity. It restores the activities of mitochondrial respiratory chain complexes I, III, IV, and V in the ileal and colonic mucosa. It also increases ATP levels, mtDNA copy number, and the expression of mitochondria‑encoded genes *COX1* (cytochrome c oxidase subunit 1) and *ND1* (NADH dehydrogenase subunit 1), suggesting that MGN‑3 promotes mitochondrial biogenesis and restores oxidative phosphorylation function. On this basis, MGN‑3 further downregulates the expression of caspase‑3, 8, 9, and 10, reducing IEC apoptosis induced by ionizing radiation. It also lowers barrier damage indicators such as endotoxin, diamine oxidase (DAO), and D‑lactate, thereby helping to maintain intestinal epithelial integrity [75]. Notably, even monosaccharides, the basic building blocks of polysaccharides, have been shown to possess similar functions. Arabinose [34] and allulose [76] prevent tight junction disruption mediated by MLCK, while also reducing intestinal epithelial barrier damage associated with mitochondrial dysfunction.

### Natural polysaccharides modulate mitochondria-organelle crosstalk to prevent and treat CRC

Beyond the intrinsic functions of mitochondria, dynamic interactions between mitochondria and other organelles such as the endoplasmic reticulum (ER) and lysosomes profoundly influence cellular stress responses, metabolic reprogramming, and cell fate determination. Between mitochondria and the ER, physical connections are primarily established through mitochondria-associated membranes (MAMs) [77]. Within these structures, the IP3R (inositol 1,4,5-trisphosphate receptor)-GRP75 (glucose-regulated protein 75)-VDAC1 (voltage-dependent anion channel 1) complex mediates Ca^2^⁺ transfer from the ER to mitochondria, which is important for maintaining mitochondrial energy metabolism [78]. Under stress conditions, ER dysfunction triggers mitochondrial Ca^2^⁺ overload via MAMs, leading to disrupted energy metabolism, excessive ROS accumulation, and opening of the mPTP, ultimately resulting in cell apoptosis [78–80]. In colitis, abnormalities in this pathway have been confirmed to be closely associated with intestinal barrier damage. Furthermore, the crosstalk between mitochondria and lysosomes also plays an important regulatory role. Lysosomes can modulate mitochondrial metabolism through Ca^2^⁺ release and membrane contact sites, and they also participate in autophagosome-lysosome fusion [81–83]. Conversely, mitochondrial functional status affects lysosomal homeostasis. For example, loss of *RAB7* (Ras-related protein Rab-7) simultaneously triggers abnormal mitochondrial division and impaired lysosomal acidification [84]. In addition, when *mTOR* is inhibited, *TFEB* (transcription factor EB) promotes lysosomal biogenesis to support mitochondrial metabolism [85, 86]. Therefore, targeting the interaction nodes between mitochondria and other organelles may represent an important pathway through which natural polysaccharides regulate CRC-related mitochondrial homeostasis.

Regarding the intervention in mitochondria-endoplasmic reticulum interactions, LNT can simultaneously affect MAMs-related Ca^2^⁺ transport and the ER stress pathway. On one hand, it activates IP3R to promote Ca^2^⁺ release from the ER, leading to mitochondrial Ca^2^⁺ overload. On the other hand, it activates the PERK (PKR‑like ER kinase) and IRE1α (inositol‑requiring enzyme 1α) signaling pathways and upregulates BIP (binding immunoglobulin protein) and CHOP (C/EBP homologous protein) expression, thereby inducing ER stress. The synergistic action of these two pathways increases the expression of Bax, cleaved-caspase-3, and LC3-II (microtubule-associated protein 1 light chain 3-II) in CRC cells, while decreasing the levels of Bcl-2 and p62 (sequestosome-1). This ultimately results in the coordinated induction of apoptosis and autophagy [87]. These findings suggest that natural polysaccharides can amplify their intervention on mitochondrial function in tumor cells by integrating MAMs-mediated Ca^2^⁺ homeostasis imbalance with ER stress responses. Unlike the aforementioned calcium‑dependent mechanisms, the *Uncaria rhynchophylla* polysaccharide (URP70-1) induces endoplasmic reticulum (ER) stress by activating the Glucose-regulated protein 78 (GRP78)/Eukaryotic translation initiation factor 2 subunit 1(EIF2S1)/CHOP pathway, which subsequently upregulates the Bax/Bcl‑2 ratio, triggers a decline in mitochondrial membrane potential, and promotes cytochrome c release, ultimately activating caspase‑9/3‑dependent apoptosis and suppressing colon cancer in CT26 cells and a zebrafish model [88]. These findings indicate that natural polysaccharides can modulate the ER‑mitochondria interplay through multiple modes—either via MAMs‑mediated Ca^2^⁺ homeostasis disruption or through activation of transcriptional pathways such as CHOP—thereby amplifying the regulatory effects on mitochondrial function.

Regarding mitochondria-lysosome interactions, the effects of natural polysaccharides show a certain context dependency. ACP upregulates *CBP* (CREB-binding protein) and enhances *TFEB* transcriptional activity, thereby promoting the expression of lysosome-related genes such as *CTSD*. As lysosomal membrane permeability increases, cathepsin D leaks out, triggering mitochondrial outer membrane permeabilization and cytochrome c release, which ultimately induces apoptosis in CRC cells [89]. Tea polysaccharides can also induce apoptosis through a similar lysosome-mitochondria crosstalk pathway [90]. Other studies have shown that even monosaccharides can regulate mitochondria-lysosome function. Arabinose [34] and allulose [76] alleviate mitochondrial dysfunction and reduce lysosomal dysfunction-induced damage to IECs by inhibiting lysosomal rupture and cathepsin B release. Overall, the functional crosstalk between mitochondria and other organelles such as the ER and lysosomes provides a new mechanistic perspective for understanding the multi-target effects of natural polysaccharides against CRC. However, current research in this area is still limited, and more direct evidence is needed to support this mechanism in the future.

In summary, the anti‑CRC effects of natural polysaccharides do not rely on a single target. Instead, they exert their actions in a coordinated manner across multiple levels centered on mitochondrial dysfunction, including the regulation of ROS homeostasis, apoptosis and autophagy, immune metabolism, intestinal epithelial barrier integrity, and organelle crosstalk. We summarize the key physicochemical characteristics of natural polysaccharides and their potential structure–activity relationships (Table 1), and outline the main mechanisms by which these polysaccharides modulate mitochondrial function to prevent and treat CRC (Table 2). Overall, current evidence supports that mitochondrial functional regulation represents a fundamental mechanistic basis for the action of natural polysaccharides against CRC. However, most of the available evidence remains preclinical, and the correspondence between structural features and functional outcomes requires further elucidation.

**Table 1:** Structural characteristics and extraction of natural polysaccharides

Classification	Name	Source	Molecular weight	Structure features	Primary composition (molar ratio)	Extraction/Modification method	References
Fungal polysaccharides	ACP	Agrocybe cylindraceae powder	156 kDa	Main chain: β-(1 → 6)-Glcp, α-(1 → 6)-Galp, α-(1 → 2,6)-Glcp (3:1:1)	Fuc:Gal:Glc = 1:4:8	95% EtOH(ethanol) defatting → hot water extraction (90 °C, 4 h) → 80% EtOH precipitation → Sevag deproteinization → dialysis → graded EtOH precipitation (20%/80%) → lyophilization	[89]
	SVP-A-1	The powder of *Sanghuangporus vaninii* fruiting bodies	22.5 kDa	Main chain: → 6)-β-Galp-(1 → 6)-β-Galp-(1 → 2,6)-β-Galp-(1 →	Fuc:Gal:Glc:Man:Fru:GlcUA = 24.47:42.94:11.89:19.99:0.64:0.07	Petroleum ether defatting → hot water extraction (80 °C, 2 h) → Sevag deproteinization → 85% EtOH precipitation → DEAE-cellulose → Sephacryl S-400 → Superdex 200	[26]
	HEFPs	The fruiting bodies of *Hericium erinaceus*	NR	β-configuration (FT-IR: 918 cm⁻^1^)	Ara:Gal:Glc:Man = 8.99:11.15:1.2:1.97	Hot water extraction (80 °C, 3 h) → K₃Fe(CN)₆/Zn(Ac)₂ deproteinization → dialysis (3.5 kDa) → 80% EtOH precipitation → lyophilization	[52]
	EP-1	Cultured mycelium of *Hericium erinaceus*	3.1 kDa	NR	NR	Hot water extraction (70 °C, 12 h) → 80% EtOH precipitation → ultrafiltration (3 K/0.2 K) → DEAE-Sephadex (H₂O elution)	[56]
	CASP	*Stropharia rugosoannulata*	11.1–97.8 kDa	β-glycosidic bond	Glc, Man, Gal	Decolorization/degreasing → sequential extraction (water → hot water → high-pressure hot water → diluted alkali → concentrated alkali) → DEAE-52 cellulose column chromatography → dialysis → lyophilization	[91]
	EPS1-1	*Rhizopus nigricans *exopolysaccharide	9.7 kDa	NR	Glc:Man:Gal:Fru = 5.89:3.64:3.20:1.00	Fermentation → ethanol precipitation → deproteinization/decolorization → ion exchange chromatography → gel filtration → lyophilization	[92]
	LNT	The dried fruit bodies of Lentinula edodes	507.2 kDa	β-(1 → 3)-D-glucan	D-glucose	Hot water extraction → EtOH precipitation → column chromatography	[87]
Plant polysaccharides	ABPA1	Aloe vera	360 kDa	→ 4)-β-D-Manp-(1 → (acetylated mannan)	Man:Glc:Gal:Ara = 89.5:6.1:3.6:0.8	EtOH precipitation → dialysis → DEAE-cellulose → gel filtration → lyophilization	[31]
	DOP	Dried Dendrobium officinale stem	312 k Da	β−1,4-linked mannopyranosyl and glucopyranosyl	Glc/Gal = 5.8 ± 0.1	Defatting → hot water extraction → EtOH precipitation → α-amylase → repeated freeze–thaw → dialysis → lyophilization	[14]
	DRP	Dandelion root	40,644 Da:23.57% 965 Da:76.43%	NR	Man:Glc:Rha:GlcUA:GlyA:GluA:Gal: Ara = 0.56:0.30:0.28:0.27:0.65:97.06:0.24:0.42	Ultrasound-assisted water extraction (700W, 40 min) → deproteinization → decolorization → EtOH precipitation → lyophilization → DEAE-cellulose → Sephadex G-100	[38]
	SDRP	Dandelion root	13.7 kDa	DS = 1.49; triple helix; sulfation at C6	Man:Gal:Glc:Ara:GlcN = 1.82:0.26:94.55:0.25:2.98	DRP (0.5 g) + H₂SO₄:n-BuOH (3:1) + (NH₄)₂SO₄ (0.154 g) at 0 °C, 91 min → pH 7–8 → dialysis → lyophilization	[41]
	MGN-3	Rice bran	30–100 kDa	Main chain: β−1,4-xylan	Rha,Fuc,Ara,Xyl,Man,Glc,Gal	Enzymatic modification of rice bran with shiitake mushroom extract	[75]
	URP70-1	*Uncaria rhynchophylla root*	8.2 kDa	Main chain: → 3,5)-α-L-Araf-(1 → and → 2,5)-α-L-Araf-(1 → Branched chain: → 3)-α-L-Araf, → 5)-α-L-Araf, → 6)-β-D-Galp	Ara: Gal: Xyl = 52.8: 35.4: 11.8	Hot water extraction → Ethanol precipitation → DEAE-FF anion exchange chromatography → Sephadex G-75 gel filtration chromatography	[88]
	PPPS	Taishan pinus massoniana pollen	25–128 kDa	NR	Man,Xyl,GlcUA, GalA, Glc, Gal, Ara,Ribose	Ultra-micro pulverization → water extraction → EtOH precipitation → DEAE-cellulose → Sephadex G-200	[93]
	CVP-AP-I	*Chuanminshen violaceum* stems and leaves	35.34 kDa	Main chain: 1,4-α-GalpA-linked homogalacturonic acid (HG) long chain	GalA:Gal:Rha:Glc:Ara:Man = 45.35:27.37:15.16:7.56:3.66:0.90	Ultrasound-assisted extraction → EtOH precipitation → dialysis → lyophilization → anion-exchange → gel filtration	[36]
	BP-1	The highland barley	67 kDa	NR	Glc:Xyl:Ara:Rha = 8.82:1.92:1.50:1.00	Hot water extraction → enzyme treatment → ethanol precipitation → centrifugation → lyophilization (crude polysaccharide) → Sepharose CL-4B column chromatography → collection of BP-1	[94]
	SPS-CF	Green alga *Capsosiphon fulvescens*	5.8 kDa	Main chain: 4-linked α-L-rhamnose-3-sulfate and β-D-xylose (ulvobiose)	Xyl: 38.6–49.4 mol%, Rha: 39.6–45 mol%, Man: 4.2–10.2 mol%, Gal: < 2.3 mol%	0.01 N HCl extraction → 75% ethanol precipitation → CaCl₂ precipitation to remove impurities → dialysis (MWCO 14 kDa) → DEAE-cellulose column chromatography (NaCl gradient) → freeze-drying	[95]
	Fucoidan	Fucus vesiculosus	1300 kDa	Main chain: → 3)fuc-2-OSO3(-)(1 → 3)fuc(1 →	Fuc:Gal:GlcUA:Glc:Xyl:Rha = 80:9:7:2:1:1	Organic defatting → ultrasound-assisted CaCl₂ extraction → CTAB precipitation → NaI dissociation → dialysis → lyophilization	[96]
		Brown seaweed *Sargassum cinereum*	NR	Main chain: α−1,3-linked or α−1,4-linked L-fucose with sulfate groups	NR	Dried seaweed milled → extraction	[97]
Microbial-derived polysaccharides	LMW-AP-FBG	Aureobasidium pullulans	NR	Main chain:β−1,3-glycosidically linked glucose units.β−1,6-glycosidically linked glucose units	β-D-glucan	Fermentation → EtOH precipitation → ball milling (6 h)	[98]
	CA	Escherichia coli	NR	Fucose-rich heteropolysaccharide	NR	Fermentation → centrifugation → concentration → dialysis (12–14 kDa) → TCA deproteinization → EtOH precipitation → dialysis → lyophilization	[99]
Animal-derived polysaccharides	ERPP	Ruditapes philippinarum	414.949 kDa	NR	Glucose	Trypsin enzymatic hydrolysis (37 °C, 3 h) → 30 kDa ultrafiltration → EtOH precipitation	[100]

These polysaccharides are derived from a wide range of sources, including fungi, plants, microorganisms, and animals. Their molecular weights span from 3.1 to 1300 kDa. Low-molecular-weight polysaccharides exhibit better cell permeability, whereas high-molecular-weight polysaccharides often require nano-delivery systems. The monosaccharide composition is dominated by glucose, mannose, and galactose. Mannose-rich polysaccharides possess immune-targeting potential, while fucose-rich polysaccharides are involved in lysosome-mitochondria crosstalk or P-selectin targeting. β-Glucans readily activate the Dectin-1/NF-κB pathway, whereas polysaccharides with an α-galacturonic acid backbone tend to exert antioxidant effects through Nrf2. Chemical modification can alter molecular weight and conformation, thereby enhancing antioxidant activity and targeting ability. The core extraction methods include hot water extraction followed by alcohol precipitation and column chromatography, with some studies employing green techniques such as ultrasound-assisted or enzyme-assisted extraction. This table summarizes the key structural parameters and preparation information of the above polysaccharides, which may serve as a reference for structure–activity relationship studies. NR: not reported; –: no data or not applicable. Ara: Arabinose; BP-1: Barley Polysaccharide-1; CA: Colanic Acid; CASP: Concentrated alkali-extracted polysaccharide; CTAB: Cetyltrimethylammonium Bromide; DEAE: Diethylaminoethyl; DRP: Dandelion Root Polysaccharides; EP-1: a polysaccharide from cultured mycelium of *Hericium erinaceus*; EPS1-1: Exopolysaccharide from Rhizopus nigricans; ERPP: *Ruditapes philippinarum* polysaccharide; EtOH: ethanol; FT-IR: Fourier-transform infrared spectroscopy; Fru: Fructose; Fuc: Fucose; Gal: galactose; GalA: Galacturonic acid; Galp: Galactopyranose; GalpA: Galactopyranuronic acid; Glc: Glucose; GlcN: Glucosamine; GlcUA/GluA: Glucuronic acid; Glcp: Glucopyranose; GlyA: Glyceric acid; HEFPs: a fruiting body polysaccharide of *Hericium erinaceus*; Man: Mannose; Manp: Mannopyranose; PPPS: A Taishan *Pinus massoniana* pollen polysaccharide; Rha: Rhamnose; SDRP: sulfated Dandelion Root Polysaccharides; SPS-CF: Sulfated Polysaccharide from *Capsosiphon fulvescens,* TCA: Trichloroacetic acid; Xyl: Xylose

**Table 2:** Mitochondria-related mechanisms of natural polysaccharides in the prevention and treatment of CRC

Name	Study model	Dose and duration	Efficacy (Quantified)	Key regulatory indicators	References
ACP	In vitro: HCT-116 (CRC)	200–400 μg/mL; 24 h	IC₅₀ = 490 μg/mL; ↑Apoptosis	↑*H3K27ac*, ↑*CTSD*, ↑*TFEB*; ↓MMP; ↑*Bax*, ↓*Bcl-2*, ↑*Caspase9/3* (Pepstatin A reverses)	[91]
	In vitro: HT-29 (CRC)	200–400 μg/mL; 24 h	IC₅₀ = 786 μg/mL	–	
SVP-A-1	In vitro: SW480, Caco-2 (CRC)	0.5–1 mg/mL; 24 h-7d	Dose-dependent ↓viability, migration, colony formation; ↑Apoptosis	↓MMP; ↑*Bax/Bad/Bid*; ↓*Bcl-2/Bcl-xL*; ↑*Caspase3/8/9* (No toxicity to normal cells)	[26]
	In vivo: *Apc*ᴹⁱⁿ/⁺ mice (spontaneous CRC)	500 mg/kg (i.g.); 9 wk	↓Tumor number/volume; ↓Angiogenesis	↑*CD4/CD8*, ↑IFN-γ/TNF-α, ↑TBX21; ↓p-AKT, ↓p-JAK1/2; Modulates gut microbiota	
HEFPs	In vitro: HCT-116, DLD1 (CRC)	0.3–0.8 mg/mL; 48 h	IC₅₀ = 0.59–0.70 mg/mL; Clony ↓70–80%; Apoptosis ↑26–30%	↑ROS; ↓MMP; ↑*Bax*, ↓*Bcl-2*; ↑*Caspase9/3* (N-Acetylcysteine reverses)	[52]
EP-1	In vitro: Caco-2 + H₂O₂	100–500 μg/mL (pretreat 24 h)	Viability 48% → 92%; Apoptosis 52% → ↓	↑SOD; ↓ROS, ↓MDA, ↑OCR, ↑ATP; ↑*Bcl-2*, ↓*Caspase3*; ↑OxPhos complexes	[56]
	In vivo: Rat (acetic acid UC)	1.2–2.5 g/kg (i.g.); 10 d	↓*TNF-α* (29%), ↓*IL-6* (31%), ↓*IL-8* (15%);	↑SOD, ↓MDA ↓*NF-κB p65*; ↑MMP	
LNT	In vitro: HT-29 (CRC)	800 μg/mL; 48 h	↓Proliferation; ↑Autophagy/apoptosis	↑*LC3-II,* ↑*Beclin1*, ↓p62; ↑*Bax/Bcl-2*, ↑*Caspase* 3; ↑*p-PERK*, ↑*p-IRE1α*, ↑*ATF4*, ↑*CHOP*; ↑Ca^2^⁺ (3-MA/CQ/4-PBA reverse)	[87]
	In vivo: NOD/SCID (HT-29 xenograft)	1–5 mg/kg (i.v., q2d); 21 d	Tumor inhibition 45–51%	Same as above (CQ/2-APB reduce efficacy)	
ABPA1	In vitro: RKO, SW480 (CRC)	200 μg/mL; 2–8 h	Time-dependent ↑apoptosis;	↓ATP; ↑ROS ↑*Bax* translocation, ↑Cytochrome c; ↑*Caspase9/3*; ↓MMP; ↓ETC I/II (RKO cells); TCA changes (Cell line-dependent)	[31]
	In vivo: Orthotopic MC38-luc (C57BL/6)	100 mg/kg (i.g.); 10 d	Day10: ↓Tumor fluorescence (p < 0.05); ↑TUNEL,	↑*Caspase*3	
DOP	In vitro: CT26 (CRC)	400–800 μg/mL; 48 h	Viability ↓40% (800 μg/mL); IC₅₀ = 868 μg/mL (72 h)	↑ROS, ↓MMP, ↓ATP, ↓OCR; ↑*LC3-II*, ↓*p62*; ↑*p-AMPK*, ↓*p-mTOR* (N-Acetylcysteine reverses)	[14]
	In vitro: IEC-6 (normal)	400–800 μg/mL; 48 h	No cytotoxicity (selective)	–	
CA	In vitro: HCT-116 (CRC)	2–256 μg/mL; 72 h	Mild ↓proliferation (80% viability at 256 μg/mL)	Mitochondrial rearrangement/clustering	[99]
ERPP	In vitro: HT-29 (CRC)	250–2000 μg/mL; 48 h	Viability < 60% (high); Colony ↓97%; Apoptosis 3% → 37%	↓*Bcl-2*, ↑*Bax*, ↑Cytochrome c, ↑*Caspase*3; ↓MMP (10.87 → 0.35); ↓ROS	[100]
	In vivo: Zebrafish (HT-29 xenograft)	31–125 μg/mL; 48 h	↓Tumor growth, migration, angiogenesis	↑Apoptosis; ↓*VEGF;* (Equivalent to 5-FU (5-fluorouracil))	
PPPS	In vitro: HCT-116, HT-29 (CRC)	400–800 μg/mL; 24–72 h	↓Proliferation; Colony ↓50% (800 μg/mL); G0/G1 arrest; ↓Migration	↑*Caspases*3/6/7/9, ↑*PARP* (poly(ADP-ribose) polymerase); ↓*MMP-9*; ↓EMT markers; TEM: Mitochondrial damage	[101]
	In vivo: BALB/c nude (HCT-116 xenograft)	400 mg/kg (i.p., 2 ×/wk); 3–6 wk	↓Tumor growth (better than 5-FU); Preventive effective; No hepatotoxicity	↑TUNEL, ↑Cleaved *caspases/PARP*; ↓Ki67	
LMW-AP-FBG	In vitro: CT26 (CRC)	1.25–10 mg/mL; 24 h	Dose-dependent ↓viability	↑*Caspa*se1/3/4/5/6/7/8/9; ↓*Bcl-xL*, ↑*Bax*; ↑Cytochrome c; ↓MMP	[35]
	In vivo: BALB/c (CT26 xenograft)	5 mg/kg/d (i.p.); 14 d	Tumor weight 1538 → 532 mg; No weight loss (5-FU causes toxicity)	–	
Polysaccharide of *Lactobacillus casei* SB27	In vitro: HCT-116 (CRC)	25–100 μM	↓Proliferation, Migration; ↑*Caspase*3/8/9 activity	↑*HINT2*, ↑*MCU*; ↑Ca^2^⁺; ↑mPTP; ↓MMP (*HINT2* overexpression enhances; *HINT2* knockdown reverses)	[53]
	In vivo: C57BL/6 (AOM/DSS CRC)	25–100 mg/kg (i.g.)	↓Tumor number/volume; ↓*IL-6*, *IL-17α*, *ptgs2*; ↑*Caspase*3/8/9	Same as above; ↓Short-chain fatty acids; Modulates gut microbiota	
Fucoidan	In vitro: HT-29 (CRC)	100–800 μg/mL; 48 h	Viability ~ 40% (800 μg/mL); Late apoptosis ~ 80%; G0/G1 ~ 50%	↑DR4, ↑*Caspase*3/6/9; ↓MMP; ↑Cytochrome c; ↑*p-JNK/JNK* (DR4 siRNA increased survival; Cytochrome c inhibitor increased survival)	[102]
	In vitro: Caco-2 human colon adenocarcinoma cells	10–1000 μg/mL; 24 h	IC_50_ = 250 μg/mL; Apoptosis ↑	MMP↓; ROS ↑;	[97]
Arabinose	In vitro: FHC + DSS	50–100 mM; 24 h	Protects lysosome; ↑Tight junctions	↑Mitochondrial mass, ↑MMP, ↓MitoROS; ↓*Cathepsin B*; ↓*p-MLC2* (CA-074 (*Cathepsin B* inhibitor)/Baf A1 reverse)	[34]
	In vivo: C57BL/6 (DSS colitis)	5–10 mg/kg (i.g.); 7 d	↓Weight loss, colon shortening; ↓Histology; ↓Permeability	↓*TNF-α, IL-6, IL-1β, MCP-1*	
	In vivo: C57BL/6 (AOM/DSS CRC)	10 mg/kg/d (i.g.); 10 wk	↓Tumor number/volume; ↓Ki-67	↓Inflammatory cytokines (CRC prevention)	
Allulose	In vitro: FHC + DSS	50–100 mM; 24 h	Same as arabinose	Same as arabinose (Ouabain/Bafilomycin A1/Rotenone reverse)	[76]
	In vivo: C57BL/6 (DSS colitis)	250–500 mg/kg (i.g.); 7 d	Same as arabinose	Same as arabinose	
	In vivo: C57BL/6 (AOM/DSS CRC)	500 mg/kg/d (i.g.); 10 wk	↓Tumor number/volume; ↓Ki-67	–	
DHP	In vitro: HCT116, Caco-2, IEC(CRC)	600 μg/mL, 24 h	HCT116 viability: 54.87% (vs BC); Caco-2 inhibition: 37%; IEC6 inhibition: 8.97%	FADD↑, Caspase-8↑, PI3K/AKT↓, Lactate ↓, UDP-GlcNAc↓, Activity of ETC complexes ↑, ROS↑, MMP↓, Bax/Bcl-2↑, Caspase-9/3↑, tBid/Bid↑	[54]
GLP	In vivo: BALB/c (AOM/DSS CRC)	200 mg/kg i.g., q.d., weeks 2–11; 9 wk	↓Tumor number	Drp1↑, OPA1↓, TOMM20↓, MMP↓, MPTP opening↑, ROS↑, ATP↓	[70]
URP70-1	In vitro: CT26(CRC)	200–800 μg/mL, 24 h	Cell viability: 50.7% inhibition at 800 μg/mL	GRP78↑, p-EIF2S1/EIF2S1↑, CHOP↑, p-ERK/ERK↑, p-p38/p38↑, ROS ↑, MMP ↑, Bax ↑, Bcl-2 ↓, Bax/Bcl-2 ↑, *Cytochrome c* ↑, cleaved-caspase-3 ↑, cleaved-caspase-9 ↑	[88]
	In vivo: Zebrafish embryo xenotransplantation (CT26 cells injected into the yolk sac)	200–800 μg/mL, 48 h	No significant difference in embryo survival rate	–	
CASP	In vitro: HT-29 (CRC)	12.5–400 μg/mL; 24–72 h	Max inhibition rate 49.08%; Dose- and time-dependent ↓ viability; ↑Apoptosis	↑Caspase-3, Bax, ↓Bcl-2	[91]
EPS1-1	In vitro: HCT-116 (CRC)	0–400 μg/mL; 24–72 h	Max inhibition 41.02% (400 μg/mL, 72 h); Apoptosis ↑;	ROS ↑, MMP↓, *Bax ↑, p53 ↑, Bcl-2 ↓*	[92]
BP-1	In vitro: HT-29 (CRC)	0–150 μg/mL 0–48 h	IC_50_ = 48.18 μg/mL Inhibition rate 71.5% (150 μg/mL; 48 h); Apoptosis rate ↑ G0/G1 arrest (59.65% → 74.06%)	↑ ROS; ↑ *p-JNK*; ↓ NF-κB *p65* nuclear translocation; ↑ Bax, ↓ Bcl-2; ↑ *Cytochrome c* release; ↑ *Caspase-8*, ↑ *Caspase-9*	[94]
SPS-CF	In vitro: HT-29 (CRC)	0–500 μg/mL 48 h	viability ↓ 64% (500 μg/mL) G2/M phase accumulation ↑ TUNEL-positive cells ↑	MMP ↓; Cleaved PARP ↑; Cleaved caspase-9/3/8 ↑; p-p38 ↑; Bcl-2 ↓	[37]
	In vivo: BALB/c nude mice bearing HT-29 xenograft	200–400 mg/kg/d 14 days(i.p.)	Tumor volume and weight: ↓ TUNEL-positive cells ↑	Cleaved PARP ↑; Cleaved caspase-9/3/8 ↑	

This table summarizes the main mechanisms by which natural polysaccharides target mitochondria to prevent and treat CRC. Modes of action: Activation of the mitochondrial apoptosis pathway (e.g., SVP-A-1, HEFPs (a fruiting body polysaccharide of *Hericium erinaceus*), ABPA1, PPPS, fucoidan, ERPP). These polysaccharides increase the *Bax/Bcl-2* ratio, promote cytochrome c release, activate *caspase-9/3*, and reduce MMP. Lysosome-mitochondria crosstalk: ACP, arabinose, and allulose induce mitochondrial dysfunction by regulating cathepsin activity. Regulation of mitophagy and ER stress: DOP and LNT act through the ROS-AMPK-mTOR or PERK/IRE1α pathways. Additionally, EP-1 improves mitochondrial bioenergetics, while CA modulates mitochondrial dynamics. In vitro and in vivo studies show dose-dependent proliferation inhibition, apoptosis induction, and tumor suppression, with low toxicity to normal cells. Key molecular validations—including N-Acetylcysteine, Pepstatin A—and immune microenvironment remodeling (e.g., SVP-A-1 increases T cells and modulates gut microbiota) support the specificity of these mechanisms. Overall, this table illustrates the network by which natural polysaccharides modulate mitochondrial function through multiple pathways and targets, providing a reference for future research. ↑: upregulation; ↓: downregulation; OE: overexpression; KD: knockdown; i.g.: intragastric; i.p.: intraperitoneal; –: not reported. 2-APB: 2-Aminoethoxydiphenyl borate; 3-MA: 3-methyladenine; 4-PBA: 4-phenylbutyric acid; AOM/DSS: Azoxymethane/Dextran sulfate sodium; ATF4: Activating transcription factor 4; BP-1: Barley Polysaccharide-1; CASP: Concentrated alkali-extracted polysaccharide; CQ: Chloroquine; DHP: *Dendrobium huoshanense* polysaccharide; DR4: Death Receptor 4; EIF2S1: eukaryotic translation initiation factor 2 subunit 1; EMT: Epithelial-mesenchymal transition; EPS1-1: Exopolysaccharide from Rhizopus nigricans; ETC I/II: Electron transport chain complex I and complex II; FADD: Fas-associated death domain protein; FHC: Fetal Human Colon cells; GLP: *Ganoderma lucidum* polysaccharide; HINT2: Histidine triad nucleotide-binding protein 2; IFN-γ: interferon-gamma; JNK: C-Jun N-terminal kinase; MCP-1: Monocyte chemoattractant protein-1; MDA: Malondialdehyde; MMP-9: Matrix metalloproteinase-9; NOD/SCID: Non-obese diabetic/Severe combined immunodeficiency; OCR: Oxygen Consumption Rate; OxPhos: Oxidative Phosphorylation; *ptgs2*: Prostaglandin-endoperoxide synthase 2; SPS-CF: Sulfated Polysaccharide from *Capsosiphon fulvescens; TBX-21*: T-box transcription factor 2; TCA: Tricarboxylic acid cycle; TOMM20: Translocase of outer mitochondrial membrane 20; UDP-GlcNAc: Uridine diphosphate N-acetylglucosamine; URP70-1: *Uncaria rhynchophylla* polysaccharide; *VEGF*: vascular endothelial growth factor A; p53: Tumor protein p53; p-p38: Phosphorylated p38 mitogen-activated protein kinase

## Natural polysaccharides target mitochondrial function to alleviate CRC drug resistance

### Mitochondrial mechanisms underlying colorectal cancer resistance

Mitochondrial dysfunction not only contributes to the development and progression of CRC but also serves as an important basis for the formation of chemotherapy resistance. Existing studies have shown that mitophagy, metabolic reprogramming, dynamics imbalance, and mtDNA abnormalities can promote CRC resistance by maintaining tumor cell survival and enhancing adaptive stress responses. Regarding mitophagy, it exhibits a biphasic role during chemotherapy. In the early stage, mitophagy helps maintain cellular metabolic homeostasis and inhibits tumor growth [103, 104]. However, during the resistance development stage, it promotes adaptive survival of tumor cells through activation of the *HMGB1/RAGE/ERK* (high mobility group box 1/receptor for advanced glycation end products/extracellular signal‑regulated kinase) pathway [72] and enhanced abnormal phosphorylation of *Drp1* at Ser616 [105]. *USP14* (ubiquitin-specific protease 14) stabilizes *BAG4* (BCL2‑associated athanogene 4) via K48 (Lysine 48)‑linked deubiquitination, thereby inhibiting Parkin‑mediated mitophagy. Knockdown of *USP14* enhances the sensitivity of CRC to oxaliplatin [106].

**Fig. 4:**
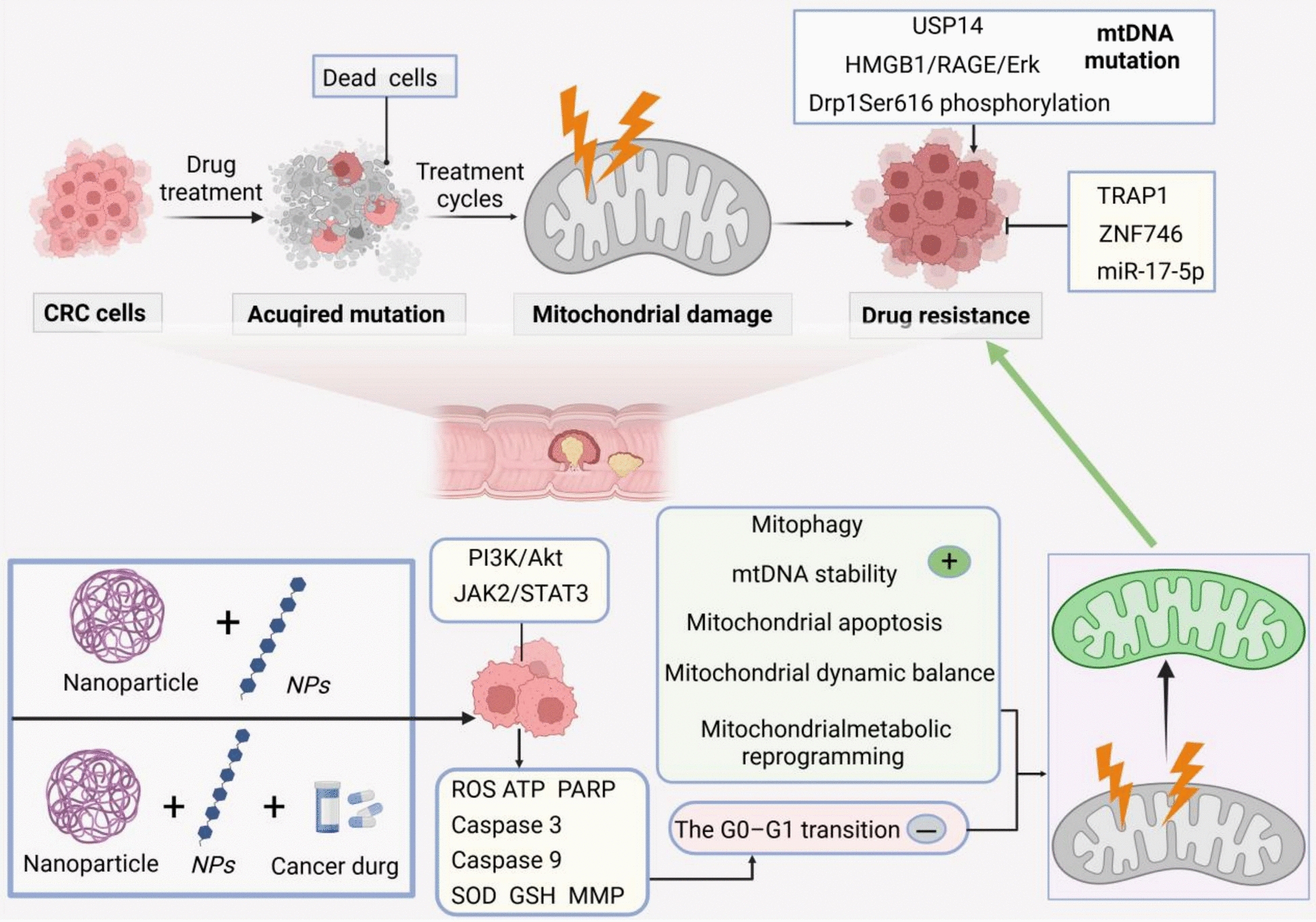
Schematic diagram of natural polysaccharide-conjugated nanoparticles targeting mitochondria to alleviate CRC resistance mechanisms. The upper left panel shows that repeated chemotherapy leads to mitochondrial damage and activates signaling pathways such as *PI3K/AKT* and *JAK2/STAT3* (Janus kinase 2/signal transducer and activator of transcription 3). This upregulates resistance‑related molecules including *USP14*, *HMGB1/RAGE/ERK*, *TRAP1*, *ZNF746*, and *miR-17-5p*, resulting in impaired mitochondrial function and exacerbated drug resistance. The upper right panel summarizes the core processes of resistance, including abnormal mitophagy, reduced mtDNA stability, impaired mitochondrial apoptosis, dynamics imbalance, and metabolic reprogramming. The lower left panel illustrates the therapeutic effects of nano‑delivery systems combined with natural polysaccharides. These effects are achieved by regulating ROS, *PARP, caspase‑3/9, SOD*, GSH (glutathione), ATP, and MMP, as well as arresting the cell cycle at the G0/G1 phase (Quiescent phase/Gap 1 phase), thereby restoring chemosensitivity. Together, this figure presents the relationships among mitochondrial dysfunction, the development of drug resistance, and the reversal of resistance through the combination of nano‑delivery systems with natural polysaccharides

In addition, mitochondrial metabolic reprogramming is another key driver of CRC resistance. This includes *TRAP1* (TNF receptor-associated protein 1)-mediated enhancement of glycolysis and altered mitochondrial serine metabolism [107], which affect the therapeutic response to oxaliplatin/irinotecan and 5-FU, respectively. In terms of mitochondrial dynamics, overexpression of *ZNF746* (zinc finger protein 746) and dysregulation of *miR-17-5p* (microRNA-17-5p) disrupt the balance of mitochondrial fusion and fission by inhibiting the expression of *MFN1/MFN2/PGC1α* (mitofusin 1/mitofusin 2/peroxisome proliferator-activated receptor gamma coactivator 1-alpha) and targeting *MFN2*, respectively, leading to resistance of CRC to 5-FU [108–110]. Meanwhile, the coding status of mtDNA directly influences mitochondrial metabolism and energy production [111]. Mutations in mtDNA can enhance cell survival by interfering with apoptotic pathways and reshaping energy metabolism [112, 113]. Notably, in MSI (microsatellite instability)-type CRC, reduced mtDNA copy number caused by *TFAM* gene mutations significantly increases resistance to cisplatin [114]. Therefore, targeting mitophagy, metabolic reprogramming, dynamics balance, and mtDNA stability may represent an important strategy to reverse CRC resistance (Fig. 4).

### Potential of natural polysaccharides in combination therapy to reverse tumor resistance

Given the central role of mitochondria in CRC resistance, natural polysaccharides can function not only as independent anti-tumor agents but also in combination with conventional chemotherapeutic drugs or other bioactive compounds. By synergistically targeting mitochondrial metabolism, redox homeostasis, and apoptotic pathways, these combinations can increase the sensitivity of tumor cells to therapy, thereby offering new strategies to reverse resistance.

The combination of natural polysaccharides with conventional anticancer drugs has been explored in several tumor types, with benefits including enhanced efficacy and reduced toxicity through mitochondrial targeting. In lung cancer, *Enteromorpha prolifera* polysaccharide combined with doxorubicin (DOX) promotes apoptosis of A549 cells, blocks G1-S phase (Gap 1 phase/Synthesis phase) transition, reduces ATP production, and inhibits mitochondrial respiration. Consequently, this combination enhances the anti-proliferative effect of DOX while reducing its toxicity and resistance [115]. In liver cancer, *Lachnum* polysaccharide combined with cyclophosphamide (CTX) exerts synergistic anti-tumor effects via the *Fas/FasL*-*caspase*-dependent death pathway and the mitochondrial apoptosis pathway, while also alleviating CTX-induced side effects [116]. Similarly, oxaliplatin combined with LNT enhances anticancer activity and reduces chemotherapy-related toxicity by strengthening mitochondrial apoptosis and suppressing *NF-κB*, *STAT3*, and *survivin* signaling [117]. In the context of CRC, LBP combined with oxaliplatin downregulates *PMI* (phosphomannose isomerase) */PI3K/AKT* signaling and inhibits the expression of the *ABCG2* (ATP-binding cassette subfamily G member 2) resistance protein in an oxaliplatin-resistant colon cancer model. This combination promotes apoptosis and reverses drug resistance, indicating that natural polysaccharides can increase the chemosensitivity of CRC cells by interfering with resistance-related survival signals and stem cell-like phenotypes [118]. Furthermore, in colorectal cancer-bearing mice treated with 5-FU, Carboxymethyl pachyman reduces mitochondrial ROS in intestinal epithelial cells, activates the *Nrf2* antioxidant pathway, and upregulates the Bcl-2/Bax ratio to inhibit the mitochondrial apoptosis pathway. It also suppresses the alteration of mitochondrial outer membrane permeabilization, thereby reducing apoptosis in normal intestinal epithelial cells. At the same time, by modulating the NF-κB/p38 inflammatory signaling pathway driven by mitochondrial ROS, Carboxymethyl pachyman protects the structural integrity of normal intestinal epithelial cells and alleviates 5-FU-induced intestinal mucositis [119]. Another natural polysaccharide from *Cibotium barometz*, increased the sensitivity of HCT15 cells to 5-FU by approximately 2.5-fold, which was associated with enhanced 5-FU-induced ROS accumulation, S-phase arrest, and apoptosis. In an orthotopic CRC model, combination with 5-FU elevated the tumor inhibition rate from 75.1% to 86.1%, while alleviating 5-FU-induced renal atrophy and tumor-induced splenomegaly [120]. These findings provide a mechanistic basis for the ability of natural polysaccharides to reduce chemotherapy-induced toxicity.

In addition to combining with conventional chemotherapeutic drugs, the synergistic effects of natural polysaccharides with other bioactive compounds offer new strategies for reversing tumor resistance. Compared with cytotoxic agents, such combinations place greater emphasis on multi‑target regulation of mitochondrial homeostasis, redox balance, and survival signaling pathways to enhance selective inhibition of tumor cells. For example, neither LBP nor anthocyanins (LRAC, *Lycium ruthenicum* anthocyanins) alone significantly inhibit tumor cell proliferation. However, when used together, they block the cell cycle at the G0/G1 phase and activate ROS‑dependent mitochondrial apoptosis by modulating *PI3K/AKT* and *JAK2/STAT3* signaling. This combination specifically inhibits the proliferation of LoVo colon cancer cells [121]. These findings suggest that natural polysaccharides and other bioactive compounds may have complementary effects on mitochondrial regulation. This provides a promising combination strategy to enhance anti‑tumor activity, reduce side effects, and delay the development of drug resistance.

Despite the potential of natural polysaccharides in reversing tumor resistance, current clinical studies remain largely focused on their use as adjuncts to chemotherapy and on symptom management. Several limitations exist, including a lack of tumor-type specificity, a limited range of polysaccharides studied, and insufficient clinical evidence directly targeting CRC resistance (Table 3). Studies have shown that APS and LNT, when used as supplements, exhibit certain therapeutic effects in the treatment of gastrointestinal tumors [122, 123]. In 2010, the China National Medical Products Administration approved GLP tablets for the treatment of leukopenia induced by radiotherapy and chemotherapy. However, relevant clinical research remains scarce. In addition, other polysaccharides such as rhamnogalacturonan I (RG-I) and soy polysaccharide fiber (SPF) have been investigated in clinical studies for anti-inflammatory effects and constipation relief [124]. Future research should strengthen pharmacokinetic and pharmacodynamic evaluations of natural polysaccharides, clarify their sensitization mechanisms, optimal combination regimens, and safety profiles when used together with chemotherapeutic agents or other bioactive compounds. Moreover, efforts should be made to advance mitochondria-targeting polysaccharide-based anti-resistance strategies into more systematic clinical validation.

**Table 3:** Partial clinical experimental research on natural polysaccharides

Name	NCT number	Study title	Study status	Conditions
APS	NCT03611712	PG2 Concurrent With Chemoradiation for Locally Advanced Esophageal Cancer	ACTIVE_NOT_RECRUITING	Cancer-related Fatigue|Survival|Tumor, Esophageal
	NCT03314805	PG2 Treatment Among Stage II/III Breast Cancer Patients Receiving Adjuvant Chemotherapy	COMPLETED	Cancer-related Fatigue|Neutropenia, Malignant
	NCT02740959	Effects of PG2 on Fatigue-Related Symptom Clusters	COMPLETED	Cancer-related Fatigue
	NCT01720550	PG2 Treatment for Improving Fatigue Among Advanced Cancer Patients Under Standard Palliative Care	COMPLETED	Cancer-related Fatigue
Genistein Concentrated Polysaccharide	NCT00584532	Effects of a Genistein Concentrated Polysaccharide for Prostate Cancer on Active Surveillance	COMPLETED	Prostate Cancer
	NCT00269555	Effects of GCP on Prostate Cancer	COMPLETED	Prostate Cancer
Sulphated fucoidan	NCT06855524	Fucoidan for Preventing Chemotherapy-Related Fatigue in Patients with Gastrointestinal or Gynecological Cancer	RECRUITING	Malignant Digestive System Neoplasm|Malignant Female Reproductive System Neoplasm
RG-I	NCT05421793	Prebiotic and Anti-inflammatory Effects of a Pectin Polysaccharide	COMPLETED	Healthy
Fructan	NCT02842281	Microbiome Fructan Metabolism and Symptoms in Childhood IBS	COMPLETED	Irritable Bowel Syndrome (IBS)
Ginseng Polysaccharide	NCT02161198	Efficacy and Safety Study of Ginseng Polysaccharide Extract	COMPLETED	Healthy
SPF	NCT01267370	Soy Polysaccharide Fiber for the Treatment of Chronic Constipation in Children: a Randomized, Double-blind Trial	COMPLETED	Constipation

Table 3 summarizes partial clinical studies on natural polysaccharides. The above studies were retrieved from ClinicalTrials.gov. The polysaccharides involved include APS, genistein concentrated polysaccharide, sulfated fucoidan, RG-I, fructan, ginseng Polysaccharide, and SPF. Regarding study focus, current clinical trials mainly concentrate on chemotherapy co-treatment, such as fatigue relief and leukopenia management, as well as symptom management, for example constipation and irritable bowel syndrome. A few studies have explored anti-tumor effects, such as in prostate cancer. Most of the studies have been completed, with only one trial on FU currently recruiting. Overall, direct clinical evidence specifically targeting CRC resistance or mitochondria-related mechanisms remains limited.

## Nano-delivery strategies and translational evaluation of natural polysaccharides targeting mitochondria

Natural polysaccharides exhibit multiple advantages in CRC prevention and treatment, including multi-target effects, low toxicity, and good biocompatibility. However, their further translation is still limited by low bioavailability, poor in vivo stability, and insufficient targeting. Nanotechnology offers innovative solutions to overcome these bottlenecks. On one hand, nanocarriers can significantly improve drug delivery efficiency. For example, exosomes modified with integrin ligands and mitochondrial tropism molecules were used to encapsulate oxaliplatin (OXA), forming a nanodrug named OXA@Exo-RD (exosome for reversing drug resistance). In CRC models, this nanodrug effectively inhibited tumor growth and metastasis by inducing ROS production and reducing ATP levels and MMP [125]. On the other hand, natural polysaccharides can serve both as therapeutic agents and as carrier materials. They enter cells through endocytic pathways such as macropinocytosis and phagocytosis [126]. When combined with targeting ligands such as *P-selectin*, mannan, or *TLR* (toll-like receptor) agonists, they achieve precise mitochondria-targeted delivery [127, 128]. This strategy not only protects the activity of polysaccharides and prolongs their circulation time but also enhances synergistic effects with existing therapies.

However, when non-endogenous materials such as metal nanoparticles are introduced into nanocarriers, the toxicological risks and clearance mechanisms following systemic absorption become another critical consideration for clinical translation. Taking silver nanoparticles (AgNPs), as an example, their systemic absorption can induce oxidative stress, reproductive toxicity, and developmental toxicity [129, 130]. The clearance and detoxification of AgNPs involve complex chemical transformations and biological regulation. First, chemical transformation of the nanoparticles occurs in vivo. AgNPs undergo accelerated oxidative dissolution in the acidic environment of the gastrointestinal tract. Silver ions, designated Ag⁺, are systemically absorbed and distributed in the form of thiolate complexes. Ag⁺ can be photoreduced to form secondary AgNPs, which are further converted to silver sulfide (Ag₂S). Through exchange reactions, Ag₂S is then converted to silver selenide (Ag₂Se) or a mixed Se/S phase. The final formation of Ag₂Se marks the immobilization and detoxification status of silver [131]. Second, altering the bonding mode of surface ligands on nanoparticles, for example by using Au-Se bonds instead of Au–S bonds, can resist glutathione-induced biotransformation in the liver. This switches the clearance route of nanoparticles from renal clearance to hepatobiliary clearance [132]. Moreover, modification with biothiols can influence the in vivo distribution and clearance kinetics of nanoparticles, thereby reducing their toxicity [133]. Furthermore, loading AgNPs into chitosan nanoparticles not only reduces their toxicity in embryonic models but may also alter their in vivo release kinetics and clearance rate [130].

These advances suggest that the translation of natural polysaccharide nanoformulations should simultaneously address both delivery efficiency and carrier safety. We have summarized the characteristics and mechanisms of action of natural polysaccharide-based nanoformulations (Table 4). The following sections will systematically describe the research progress in combining natural polysaccharides with nanotechnology from five aspects: nanocarriers, nanotherapeutics, nano-immunomodulators, mitochondrial targeting mechanisms, and pharmacokinetic/pharmacodynamic (PK/PD) translational evaluation. Special attention will be given to the specific evidence in CRC models.

### Natural polysaccharides combined with nanotechnology synergistically induce mitochondrial apoptosis

Among the research findings combining natural polysaccharides with nanotechnology, the most widespread application is their use as stabilizers, targeting ligands, or mucoadhesive promoters in nano-delivery systems. This indirectly enhances the activation of the mitochondrial apoptosis pathway by drugs, thereby exerting anti-CRC effects [134, 135]. For example, FU has good anticancer properties and recognizes *P-selectin*, a protein highly expressed on colon cancer cells [128, 136]. In one study, FU was combined with chitosan (CS) and functional polymeric nanoparticles (NPs) to form a sorafenib-targeted nanoformulation called SOR (sorafenib)-CS-FU-NPs [137]. In another study, fucoidan-coated polymeric nanoparticles based on methoxy poly(ethylene glycol)-poly(lactic acid) (mPEG-PLA-NPs) were used to encapsulate epirubicin (Epi), resulting in mPEG-PLA-FU-Epi-NPs [138]. Both nanoformulations showed high cytotoxicity and high cellular uptake in CRC cell lines in vitro. Apoptosis assays using JC-1 (tetraethylbenzimidazolylcarbocyanine iodide) dye and DCFH-DA (2′,7′-dichlorodihydrofluorescein diacetate) suggested that the enhanced cancer cell killing ability of these nanoformulations may come from greater ROS generation and stronger disruption of MMP. This leads to better activation of the mitochondrial apoptosis pathway. Another study used FU to load irinotecan (IRT) and synthesized FU/CS/IRT NPs. In vitro experiments clearly showed that this nanoformulation increased the expression of *caspase-3*, *caspase-9*, and *PARP* in HCT116 cells. It also exhibited a synergistic effect, with a combination index below 1. The anticancer activity was superior to that of IRT alone, although the specific signaling mechanisms were not elucidated [128].

Collectively, these studies indicate that natural polysaccharide-based nanocarriers can amplify the intervention efficiency on the mitochondrial damage network. This amplification is achieved through a multi-stage delivery process. The process involves tumor accumulation, receptor-mediated uptake, intracellular trafficking, and local exposure to mitochondria. However, the underlying mechanisms and subcellular evidence still need further investigation.

### Natural polysaccharides combined with nanotechnology directly promote tumor cell apoptosis and autophagy

Natural polysaccharides can not only serve as carriers to synergize with anticancer drugs but also act alone as nanotherapeutics. By being formulated into nanoparticles, they enhance anticancer efficacy while exhibiting unique synergistic effects.

One group of researchers constructed EPS-SeNPs, which are EPS-conjugated selenium nanoparticles (SeNPs). These nanoparticles were synthesized by reducing selenite ions (SeO₃^2^⁻). The resulting EPS-SeNPs showed enhanced stability and antioxidant capacity due to the formation of CO-Se bonds. They efficiently scavenged O₂•⁻ (superoxide anion radical) and ABTS⁺ (2,2′-Azino-bis(3-ethylbenzothiazoline-6-sulfonic acid) radical cation) radicals [27, 139]. The gastrointestinal release profile of EPS-SeNPs suggests that they may act as an anticancer adjuvant for targeted selenium supplementation [140]. In addition, EPS-SeNPs significantly inhibited the proliferation of HepG2 liver cancer cells by activating both intrinsic and extrinsic apoptotic pathways [18]. These nanoparticles were taken up by Caco-2 cells through clathrin- and caveolae-dependent endocytosis. Their bioactivity depended on selenium content, i.e., the Se/EPS ratio, and particle size. Specifically, EPS-SeNPs with a high selenium ratio (EPS/Se = 3/4) and a small particle size (79 nm) exhibited stronger cellular uptake [18, 141]. This finding suggests that the anti-CRC potential of EPS-SeNPs deserves further investigation. Selenium nanoparticles stabilized with Longan polysaccharide (LP), designated LP-SeNPs [142], dose-dependently inhibited proliferation in HCT-116 and HT-29 colon cancer cells. They induced ROS elevation, loss of MMP, upregulation of Bax, cytochrome c, and cleaved *caspase-3*, and downregulation of *Bcl-2*, thereby activating the mitochondrial apoptosis pathway. Notably, LP-SeNPs showed no toxicity to normal IEC-6 cells. Another study on *Tussilago farfara* polysaccharide silver nanoparticles (FFP@AgNPs) demonstrated that these nanoparticles inhibited HT-29 cell proliferation and induced apoptosis by reducing ATP levels, disrupting mitochondrial function, and damaging cell membrane integrity [143]. In addition, pomegranate polysaccharide nanoemulsion (PGPs-NE) achieved a high encapsulation efficiency of 92.82% through oil-phase self-emulsification technology. This nanoemulsion exhibited synergistic antioxidant, anti-inflammatory, and anti-tumor effects in CRC cells [144].

### Natural polysaccharides combined with nanotechnology enhance anti-tumor immune function

Natural polysaccharides possess immunostimulatory properties, low toxicity, and high safety. When combined with nanotechnology, they can further enhance both humoral and cellular immunity, thereby reshaping the tumor immune microenvironment [145]. Studies have shown that polysaccharide-modified nanovaccines can precisely target DCs. By activating pattern recognition receptor pathways, they significantly enhance antigen presentation efficiency, which in turn triggers potent T cell immune responses.

In the area of nanomedicines, researchers have developed a nano-delivery system based on AS, designated AS-PP (peptide)-DOX. In this system, AS not only serves as a carrier for targeted delivery of DOX but also modulates the tumor microenvironment. It increases the pro-inflammatory cytokine *IL-2*, reduces the immunosuppressive cytokine *IL-10*, restores the Th1 (T helper 1)/Th2 (T helper 2) balance, and enhances anti-CRC tumor immunity [146]. Another nanoparticle formulation, the lentinan-ursolic acid (LNT-UA) nanoparticle, exhibits good stability and high drug loading capacity. It was prepared by nanoprecipitation to improve the solubility of UA and induce immunogenic cell death (ICD), thereby activating systemic anti-tumor immunity. Moreover, LNT-UA combined with anti-CD47 antibody (αCD47) enhances macrophage phagocytosis, suppresses CRC, and prolongs survival [147].

In addition to nanomedicines, various innovative polysaccharide-based nanovaccines have also shown favorable immune activation effects. In the mannan core–shell nanovaccine designated Mannan-PLA-PEI (polyethylenimine), mannan acts as a *TLR4* agonist. It works synergistically with *CpG* (cytosine-phosphate-guanine oligodeoxynucleotide), a *TLR9* agonist, to enhance dendritic cell activation and lymph node targeting. This nanovaccine achieved a 50% complete cure rate in a pan-cancer model [148]. The LBP-modified *CD155* nanovaccine (LBP-CD155L NVs) promotes dendritic cell maturation through the dual *TLR4*/MGL (macrophage galactose-type lectin) pathways. It enhances CD8⁺ T cell cytotoxicity and suppresses immunosuppressive cells such as MDSCs (myeloid-derived suppressor cells) and regulatory T cells. Furthermore, combining this nanovaccine with an anti-*PD-1* antibody further promotes T cell infiltration and enhances the immunotherapeutic effect [149]. Another nanovaccine, NP-TCL@APS, encapsulates tumor cell lysate (TCL) within APS nanoparticles. It activates DCs via the *MHC* (major histocompatibility complex) class I and II pathways, enhances antigen cross-presentation, and induces potent CD8⁺ and CD4⁺ T cell responses. This vaccine may further optimize dendritic cell function through PRRs such as *TLR4* [17].

**Table 4:** Application research on the combination of natural polysaccharides and nanotechnology

Nanocomposite drug name	Characterization				Target	Bioactive effects	References
	Particle size (nm)	Potential (mV)	Encapsulation rate (%)	PDI			
PGPs-NE	9.5	− 30.6	92.82	< 0.2	ROS	Antioxidant Anti-inflammatory Anti-tumor	[144]
SOR-CS-FU-NPs	209.98 ± 1.25	29.24 ± 3.74	83.22 ± 3.31	0.229 ± 0.022	ROS MMP	Targeted delivery of SOR Cellular uptake ROS production Mitochondrial damage	[112, 137]
mPEG-PLA-FU-Epi-NPs	98.28 ± 1.44	− 27.60 ± 0.5	89.23 ± 2.71	NR	*VEGF* MMP-9 ROS MMP	Synergistic anti-cancer Reduces cardiotoxicity	[138]
FU/CS/IRT NPs	230.8 ± 2.5	26.7 ± 1.2	66.8 ± 0.2	≤ 0.3	*Caspase 3* *caspase8* *caspase9* *PARP* Cytochrome c *P53*	Synergistic cytotoxicity Promote apoptosis	[128]
EPS-SeNPs	80–150	NR	NR	NR	O₂•⁻ ABTS⁺ ROS	Control selenium release Antioxidant Induce apoptosis	^27,140,141,150^
FFP@AgNPs	69.79	− 17.1	NR	0.217	Nicotinic acid Nicotinamide GMP AMP	Cell proliferation Mitochondrial dysfunction Cell membrane damage	[143]
AS-PP-DOX	129.00 ± 3.32	− 28.45 ± 0.22	NR	0.119	Th1/Th2	Chemotherapy-immune synergy	[146]
LNT-UA	68.8	− 18.3	39.4 ± 0.7	0.177	ICD DC Macrophages	Immune microenvironment	[147]
LNT-SeNPs	59	NR	NR	NR	*p21* *p27* *caspase-9* *caspase-3* Bax/Bcl-2 MMP	Mitochondrial apoptosis	[16]
NP-TCL@APS	249.17 ± 1.54	− 11.3 ± 0.138	78.22 ± 6.34	0.141 ± 0.007	DC CD8+ CD4+ *TNF-α* *IFN-γ* *GZMB*	Anti-CRC	[17]
MPVax-CpG/OVA	110 ± 20	11 ± 2	NR	NR	DC	Antigen cross-presentation	[148]
LBP-CD155L	165.96 $\pm$ 18.34	1.5 $\pm$ 0.3	55.4 $\pm$ 2.1	0.17 $\pm$ 0.05	MDSCs Macrophages CD8+ T	Immune microenvironment	[149]
CHH-T/NPs	132.5 ± 2.1	− 12.03 ± 1.2	NR	NR	MCT1 Lactate ATP *LC3Ⅱ/Ⅰ* *p62*	Blocks protective autophagy Suppresses cancer stemness	[151]
IN-LA CR micelles	170–260	− 13.76 ± 1.20	56.6	NR	GSH MMP *Caspase-3*	Induces apoptosis Inhibits proliferation	[152]
RSNM	172.3 ± 2.1	− 18.2 ± 0.15	56.03 ± 1.0	0.237 ± 0.004	MMP	Induces apoptosis Inhibits proliferation	[153]
ACN/CH/CS NPs	350.11 ± 0.99	+ 42.55 ± 0.54	86.32 ± 0.15	0.158 ± 0.03	Loss of mitochondrial cristae Disruption of bilayer membrane	Induces apoptosis Induces mitochondrial structural damage	[154]
GER-BSA-DEX-F NPs	117.8 ± 4.39	− 10.28 ± 0.42	74.95 ± 5.38	0.188 ± 0.02	ROS MMP G2/M arrest F-actin fragmentation DNA damage	Induces apoptosis Inhibits proliferation Disrupts cytoskeleton	[155]
CNC/5FU	119.25 ± 2.3	− 38.10 ± 1.7	83.50 ± 1.52	NR	MMP Mitochondrial membrane damage Apoptotic activity	Induces apoptosis Inhibits clonogenicity	[156]
t-car-γ-Fe₂O₃	200–550	− 7.70 ± 2.8	NR	NR	ROS MMP *Caspase-3* Bcl-2 Bcl-xL *XIAP* *PARP-1*	Induces apoptosis Inhibits proliferation	[157]

Table summarizes representative nanoformulations based on natural polysaccharides for the treatment of CRC. The formulations include polysaccharide-conjugated nanoparticles, polysaccharide-coated drug delivery systems, and polysaccharide-based nanovaccines, among others. The main biological effects include the induction of mitochondrial apoptosis, characterized by elevated ROS levels, loss of MMP, and activation of *caspases* and *PARP*. Additional effects include anti-inflammatory and antioxidant activities, immunomodulation such as dendritic cell activation, T cell responses, and suppression of MDSCs, as well as synergistic anti-tumor effects when combined with chemotherapy or immunotherapy. AMP: Adenosine monophosphate; GMP: Guanosine monophosphate; *GZMB*: Granzyme B; *IFN-γ*: interferon-gamma; *p21*: Cyclin-dependent kinase inhibitor; *P27*: Cyclin-dependent kinase inhibitor 1B; *P53*: Tumor protein p53; *VEGF*: vascular endothelial growth factor A. CHH-T/NPs: α-cyanohydroxycinnamic acid (CHC)-hyaluronic acid (HA)-hydroxychloroquine sulfate (HCQ) polymer prodrug nanoparticles co-assembled with mitochondria-targeting IR820; T820: Mitochondria-targeting IR820 derivative; IR820: New Indocyanine Green, a near-infrared dye; MCT1: Monocarboxylic acid transporter 1; IN-LA CR micelles: Crosslinked inulin‑lipoic acid micelles; RSNM: Resveratrol‑loaded inulin‑pluronic F68‑stearic acid nanomicelles; ACN/CH/CS NPs: Black rice anthocyanins‑loaded chondroitin sulfate/chitosan nanoparticles; GER-BSA-DEX-F NPs: Folate receptor‑targeted dextran‑modified geraniol‑bovine serum albumin nanoparticles; CNC/5FU: 5‑Fluorouracil‑loaded cellulose nanocrystals; t-car-γ-Fe₂O₃: Iota‑carrageenan‑gamma‑maghemite nanocomposite; *XIAP*: X-linked inhibitor of apoptosis protein

### Mechanisms of mitochondrial targeting by natural polysaccharide nanoformulations

Although the studies mentioned above commonly attribute the anti-tumor effects to mitochondrial dysfunction, a core mechanistic question remains insufficiently addressed: how do nano-systems achieve mitochondrial targeting? Based on current evidence, natural polysaccharide nano-systems may regulate mitochondrial function through at least three possible mechanistic pathways. These pathways differ in their evidence strength and modes of action.

The first mechanism is active targeting, which mainly relies on the modification of mitochondrial targeting ligands and hierarchical response design. The most commonly used ligand is triphenylphosphine [158], followed by cell-penetrating peptides (CPPs), and mitochondrial targeting signal peptides (MTS) [159]. To achieve active mitochondrial targeting, researchers have conjugated lipophilic cationic groups such as triphenylphosphine (TPP) or berberine to polysaccharide backbones. However, cationic carriers are easily cleared in circulation and can be toxic. To address this issue, Fang et al. [160] designed a pH-responsive charge-reversal nanoparticle, named AS-PBA/GA-CDB@Cur, in a pancreatic cancer model. The core of this nanoparticle consists of CS oligosaccharide designated GA-CDB (Gallic acid-chitosan oligosaccharide-dithiopropionate acid-berberine), which contains disulfide bonds and a mitochondrial targeting group, berberine. The shell is composed of AS conjugated with phenylboronic acid (AS-PBA). The core and shell are linked through borate ester bonds. Under physiological pH, the surface of AS-PBA/GA-CDB@Cur is negatively charged, which prolongs circulation time. Upon entering the acidic tumor microenvironment, the shell detaches and the core becomes positively charged. This allows the nanoparticle to target mitochondria via berberine, and drug release is triggered by glutathione. Guo et al. [161] employed HA and dextran micelles in a breast cancer model. They utilized *CD44* targeting and hyaluronidase-responsive exposure of TPP, while using a ROS-sensitive bond to release the drug. Direct evidence for the above active targeting strategies in CRC remains limited. For example, Xu et al. [162] used HA-modified liposomes to co-deliver the chemotherapeutic agent RA-XII (rubia yunnanensis cyclopeptide XII) and the mitochondria-targeting photosensitizer TPPP (protoporphyrin-IX attached with triphenylphosphonium). In colon cancer HCT116 cells and tumor-bearing mice, they demonstrated that HA-mediated *CD44* targeting enhanced tumor uptake. TPPP localized to mitochondria and generated ROS upon laser irradiation, synergistically inducing apoptosis and inhibiting tumor growth. However, studies combining natural polysaccharide-based active targeting with mitochondrial targeting ligands are still rare in CRC. More validation using various models is needed to provide direct evidence.

Unlike active targeting, some nanoparticles do not require ligand modification and can act on the mitochondrial surface through physical means, a mechanism termed surface action. Some small-sized nanoparticles or polysaccharide oligosaccharide fragments released from nanoparticles may directly regulate the opening of mPTP and ROS release through physical contact or by binding to outer mitochondrial membrane proteins, the voltage-dependent anion channel [163]. EPS-SeNPs [18] and FFP@AgNPs [143] may also function through this mechanism in CRC. However, direct imaging evidence demonstrating the co-localization of these nanoparticles with mitochondria is still lacking in relevant studies.

Finally, the third mechanism is indirect regulation, specifically the lysosome-mitochondria crosstalk pathway. Most natural polysaccharide nanoparticles are primarily retained in lysosomes after entering cells via endocytosis. The polysaccharides or their degradation products can induce lysosomal membrane permeabilization. This leads to the leakage of cathepsins such as CTSD, encoded by the gene *CTSD*, into the cytosol. These cathepsins then cleave the mitochondrial outer membrane protein *Bid* or activate *Bax*, triggering mitochondrial outer membrane permeabilization (MOMP), and the release of cytochrome c. It should be noted that direct evidence for natural polysaccharide nanoparticles functioning through this pathway is currently limited. The biological basis of this pathway mainly comes from studies on free polysaccharides. As described in Chapter 4, ACP and tea polysaccharides induce lysosome-mitochondria crosstalk-mediated apoptosis [89, 90]. Whether nanoformulations also rely on this pathway and to what extent remains to be further verified. Importantly, this pathway may not require nanoparticles to enter mitochondria directly; instead, it indirectly regulates mitochondrial function through inter-organelle communication.

Most existing studies infer mitochondrial targeting indirectly from downstream effects such as decreased MMP and cytochrome c release. However, in current research using orthotopic CRC models, few studies have simultaneously employed confocal co‑localization imaging or quantitative detection after subcellular organelle isolation. As a result, direct evidence for the spatial co‑localization of natural polysaccharide nanoparticles with mitochondria remains insufficient.

### Pharmacokinetic and pharmacodynamic translational evaluation of natural polysaccharide nanoformulations

The above mechanistic pathways reveal how nanoformulations affect mitochondrial function after entering cells. However, whether these mechanisms can fully exert their effects in vivo first depends on whether the nanoformulations can be effectively absorbed via the oral route and maintain sufficient systemic exposure.

Orally administered nanoformulations face multiple sequential barriers in the gastrointestinal tract. First, the chemical barrier includes the strongly acidic gastric environment at pH 1.0 to 3.0 and various digestive enzymes such as pepsin, trypsin, lipase, and pancreatic enzymes. These factors can degrade acid-labile drugs and the nanocarriers themselves, with enzymatic degradation typically being the dominant factor limiting oral bioavailability [164]. Second, the intestinal mucus layer consists of a network of cross-linked mucin with pore sizes smaller than approximately 200 nm. This mesh captures and eliminates nanoparticles through steric hindrance, charge adsorption, and rapid turnover with a renewal cycle of 47 to 270 min [165, 166]. Third, tight junctions in the intestinal epithelial barrier have pore sizes smaller than 1 nm, which strictly restrict the paracellular transport of macromolecules larger than 700 Da. Meanwhile, efflux pumps such as *P-glycoprotein* actively transport drugs back into the intestinal lumen, reducing intracellular accumulation [167]. Finally, even after successful cellular uptake, the endocytic pathway determines the intracellular fate of nanoparticles. Nanoparticles internalized via clathrin-mediated or macropinocytic pathways are prone to lysosomal degradation, whereas those taken up through the caveolae-mediated pathway can escape degradation and achieve transcytosis [168]. Therefore, nanoformulations must overcome all of the above barriers to achieve efficient oral delivery. Whether they can truly improve the in *vivo* exposure and tissue distribution of drugs is the core question in evaluating their translational potential.

In this context, although existing studies have demonstrated promising application prospects for natural polysaccharide nano-systems in terms of formulation development, in vitro activity, and in vivo tumor suppression, proving that nanoformulation is more effective is not sufficient for clinical translation from a translational medicine perspective. For natural polysaccharide-based nano-systems, more critical questions need to be addressed: Does nanoformulation truly improve their in vivo exposure, tissue distribution, and subcellular delivery efficiency? And do these pharmacokinetic changes ultimately translate into quantifiable pharmacodynamic enhancement?

The oral bioavailability of natural polysaccharides is generally low. Studies have shown that nanoformulation may improve this situation. Liu et al. [169] reported that oral delivery of curcumin (CUR) using HA-zein nanoparticles, designated HA-Zein-CUR, in nude mice bearing subcutaneous CRC xenografts increased the peak plasma concentration (Cmax) by approximately threefold compared with free curcumin. The area under the plasma concentration–time curve (AUC) increased by 9.18 times. Moreover, the accumulation of curcumin in colorectal tumor and colon tissues was significantly enhanced. Zhao et al. [170] further confirmed in an orthotopic CRC model that after oral administration of HA-trimethylamine N-oxide-coated nanoparticles (HTPBD), the fluorescence signal at the tumor site peaked at 12 h and persisted for up to 24 h. The tumor fluorescence intensity at 24 h was 1.97 times that of the unmodified control group, and the nanoparticles were mainly enriched in colorectal tumor tissue and the colon. However, current data on the pharmacokinetics and pharmacodynamics of polysaccharide nano-systems for genuine mitochondrial targeting in CRC models are limited. Fang et al. [160] evaluated charge-reversal mitochondria-targeting nanoparticles in pancreatic cancer-bearing mice. They showed that the tumor retention time could reach 24 h, and the tumor inhibition rate was 58.6%. Nevertheless, they did not directly quantify drug concentrations in mitochondria.

Overall, nanoformulation is expected to improve dispersion and stability, prolong circulation half-life, enhance local retention in the colon and tumor accumulation, and promote drug delivery to regions adjacent to mitochondria. These improvements may amplify pharmacodynamic responses such as ROS accumulation, loss of MMP, autophagy imbalance, and immune activation. However, most current reports still focus primarily on parameters such as particle size, encapsulation efficiency, cellular uptake, cytotoxicity, and endpoint tumor inhibition rates. Systematic analyses of classical pharmacokinetic parameters, tissue distribution profiles, mitochondrial co-localization efficiency, and the PK/PD relationship remain limited.

## Discussion and prospects

The role of natural polysaccharides in CRC prevention and treatment should not be viewed merely as direct inhibition of tumor cells, but rather as a systemic, multi-level regulatory process. Current evidence indicates that natural polysaccharides directly influence tumor fate by regulating ROS homeostasis, inducing mitochondrial apoptosis and autophagy, and reshaping tumor cell energy metabolism. At the same time, they improve mitochondrial function in immune cells, maintain mitochondrial homeostasis in IECs, and protect intestinal barrier integrity. Through these actions, they form a synergistic regulatory network linking the gut microbiota, local immune microenvironment, and tumor progression. Thus, mitochondria can be considered a central hub connecting microbiota, barrier function, immunity, and tumor cell fate, rather than simply an organelle for apoptosis execution. This perspective elevates the anti-CRC effects of natural polysaccharides from a single-activity observation to a systematic multi-target regulatory framework, which is more aligned with current trends in tumor biology and translational medicine. Recently, Bentharavithana et al. [171] systematically reviewed the anti-colon cancer mechanisms of various medicinal mushroom polysaccharides, covering anti-proliferation, cell cycle arrest, anti-inflammation, antioxidant effects, and apoptosis induction. However, that review did not address the specific application of nano-delivery technology in mitochondrial targeting. The present review builds upon that work by further focusing on the strategies and translational evaluation of natural polysaccharides combined with nanotechnology for mitochondrial targeting.

Despite the considerable progress made in recent years on natural polysaccharides for CRC prevention and treatment, several limitations remain in the current body of evidence.

First, regarding the structural characteristics and anti‑CRC functional activities of natural polysaccharides, as summarized in Table 1, several original studies failed to comprehensively report critical structural parameters, including molecular weight, monosaccharide composition, glycosidic linkages and branching, and chemical modifications. This limitation substantially hampers in‑depth structure–activity relationship analysis. Consequently, we are unable to definitively establish causal links between structural variations and functional outcomes, leaving most conclusions at a qualitative level. It has been recognized that in polymer science, issues such as limited datasets, broad molecular weight distributions, and incomplete structural characterization pose significant barriers to the application of machine learning [172]. Nevertheless, the rational design of therapeutic polymers relies on quantifiable predictive models—such as multiple regression and machine learning—which require complete numerical structural descriptors [173, 174]. For instance, the integration of machine learning with coarse‑grained molecular dynamics simulations can establish monomer sequence–structure–function relationships; however, this approach is contingent upon the availability of high‑quality, feature‑complete training datasets [174]. To address data incompleteness and structural heterogeneity, polymer informatics and glycoinformatics have introduced innovative strategies. Active learning enables multi‑objective optimization using sparse experimental data; with as few as 20 data points, it can achieve synergistic optimization across multiple performance parameters with a relative error below 12% [175]. Graph neural networks can decipher polymer structure–property relationships, although data quality remains a critical bottleneck [176]. Multimodal graph neural networks, which integrate molecular structure data with textual information, have been shown to reduce prediction errors by 8.6% while offering functional‑group‑level interpretability, thereby facilitating the decoupling of structure–activity relationships [177]. These emerging computational tools collectively provide a methodological foundation for the rational design and functional prediction of natural polysaccharides. Therefore, future studies reporting novel natural polysaccharides or related polymers should prioritize the provision of comprehensive characterization parameters and the curation of high‑quality structure–performance datasets. Such efforts will accelerate the transition of this field from qualitative description toward quantitative prediction.

For mitochondria-targeting nano-systems, the core issue is no longer whether they possess activity, but rather whether the contribution of mitochondrial targeting has been rigorously proven, whether the delivery advantages can be quantified, and whether the translational potential is reproducible [178, 179]. Among natural polysaccharide-based nanoformulations, chitosan is the sole polysaccharide that indirectly participates in mitochondrial targeting through its cationic properties. Its surface positive charge (approximately + 30 mV) electrostatically attracts the highly negative inner mitochondrial membrane (− 150 to − 200 mV), facilitating drug enrichment at mitochondria and amplifying the collapse of mitochondrial membrane potential as well as ROS generation [180, 181]. In contrast, anionic or neutral polysaccharides—such as hyaluronic acid, pectin, and alginic acid—lack this intrinsic capability, and their mitochondrial effects predominantly depend on the direct actions of photodynamic agents, chemotherapeutic drugs, or metal ions [182–185]. However, most existing studies infer charge-mediated mitochondrial targeting indirectly through downstream indicators such as JC-1 dye and ATP levels, without providing direct imaging evidence of nanoparticle co-localization with mitochondria or quantitative measurements of subcellular drug concentrations

As described in Section"Mechanisms of mitochondrial targeting by natural polysaccharide nanoformulations", direct evidence for active targeting strategies in CRC is still limited. For surface action and indirect regulatory pathways, direct imaging evidence demonstrating nanoparticle co-localization with mitochondria is lacking. Section "Pharmacokinetic and pharmacodynamic translational evaluation of natural polysaccharide nanoformulations" further points out that in vivo pharmacokinetic parameters and studies on the dose–effect relationship between pharmacokinetics and pharmacodynamics are extremely scarce. At this stage, there is an urgent need to move from correlative descriptions to causal validation. A complete evidence chain should be established, linking formulation parameters to mitochondrial exposure and then to therapeutic endpoints, rather than simply adding new formulation cases.

Future research should be directed toward nano-translation by establishing an integrated evaluation system that covers colon-specific release, tumor accumulation, intracellular delivery, mitochondrial localization, and pharmacokinetic and pharmacodynamic responses. This will help avoid equating general drug delivery advantages with true mitochondrial targeting. At the same time, clinical translation should be advanced with concurrent assessments of long-term safety, immunogenicity, gut microbiota disturbances, and the feasibility of large-scale formulation production. Attention should also be paid to the impact of individual differences on the response to polysaccharide nano-systems.

On this basis, new perspectives such as circadian rhythm and lipid metabolism also warrant further exploration. Circadian rhythm disruption is an important environmental risk factor for CRC that cannot be ignored. Mitochondrial dysfunction may exacerbate circadian rhythm disturbances by affecting clock genes. Studies have shown that circadian rhythm disruption leads to reduced expression of the clock genes *NR1D1* (nuclear receptor subfamily 1 group D member 1) and *BNIP3*, which inhibits mitophagy and further aggravates mitochondrial dysfunction and intestinal barrier damage [186]. In contrast, LNT can modulate the amplitude of clock gene oscillations, peach polysaccharide restores *Cry2* (cryptochrome 2) and *Per3* (period circadian regulator 3) expression by improving gut microbiota dysbiosis, and β-glucan restores the rhythmic expression of *Bmal1* (brain and muscle *ARNT*-like 1), *Clock* (circadian locomotor output cycles kaput), and *Cry1* (cryptochrome 1) in the colon [187–189]. Thus, it is worth investigating whether natural polysaccharides can interfere with the transformation from inflammatory bowel disease to CRC through the *NR1D1*-*BNIP3*-mitophagy axis. Furthermore, mitochondria serve as the central site of lipid metabolism. Inhibition of *UGT8* (uridine diphosphate-glycosyltransferase 8) can disturb sulfatide metabolism, induce mitochondrial failure, and enhance apoptosis sensitivity [190]. When inducing apoptosis, natural polysaccharide nanoformulations display a phenotype similar to that of modulating nicotinamide adenine dinucleotide (NAD⁺) metabolism [143]. Whether natural polysaccharides reshape lipid distribution to lower the apoptotic threshold by interfering with key metabolic enzymes such as UGT8, or whether they affect NAD⁺ metabolism as a central hub connecting energy homeostasis and cell survival, remains to be experimentally verified.

We may also extend the research perspective from single polysaccharides to traditional Chinese medicine formulas, which could open new avenues with greater potential for clinical translation. Take the classic formula Yiyi Fuzi Baijiang Powder as an example. It has shown potential in preclinical and clinical studies for the prevention and treatment of CRC [191, 192]. The constituent herbs of this formula contain polysaccharides with diverse activities: Coix seed polysaccharide, *Aconitum carmichaelii* polysaccharide, and *Patrinia scabiosaefolia* polysaccharide exhibit anti-tumor, immunomodulatory, anti-inflammatory, and antioxidant properties, respectively [193–195]. Based on the above, we propose the following hypothesis: Can these functionally distinct polysaccharides form a synergistic network that clears dysfunctional mitochondria at the precancerous stage by regulating the circadian rhythm-metabolism-autophagy axis, thereby blocking the malignant transformation from inflammatory bowel disease to colorectal cancer?

Overall, research on natural polysaccharides for colorectal cancer is moving from single-activity observations toward an integrated framework that combines structural optimization, microbiota regulation, precision delivery, and subcellular organelle intervention. With mitochondria serving as a key hub, natural polysaccharides exhibit multiple functions including direct anti-tumor effects, immunomodulation, barrier protection, and metabolic remodeling. This reflects a multi-target regulatory characteristic distinct from traditional single-target drugs. Future efforts should establish a tighter evidence loop linking structural characterization, mechanisms of action, delivery pathways, and translational evaluation. New directions such as circadian rhythm regulation, lipid metabolism, and formula-based synergies should also be actively explored. On this basis, natural polysaccharides and their mitochondria-targeting nano-systems hold promise as novel strategies for CRC prevention and treatment. However, their clinical value still requires further validation.

## Conclusion

Natural polysaccharides can prevent and treat CRC by targeting mitochondrial function. In terms of nano-delivery, their mitochondrial targeting intervention mainly relies on three pathways: ligand-modified active targeting, physical surface interaction, and indirect lysosome-mitochondria crosstalk. Natural polysaccharides can also reverse tumor resistance by interfering with mitophagy, metabolic reprogramming, and dynamics imbalance in combination with chemotherapeutic drugs. Their biological activities are collectively influenced by structural features including molecular weight, monosaccharide composition, glycosidic bond type, branching structure, and chemical modification. Natural polysaccharides and their mitochondria-targeting nano-systems show promise for the prevention and treatment of CRC. However, in vivo evidence of nanoparticle co-localization with mitochondria and the pharmacokinetic-pharmacodynamic translational evaluation system still need improvement. Therefore, future research should focus on standardized structural characterization, in-depth mechanistic validation, and integrated evaluation of delivery and efficacy. Further attention should be given to the unique challenges of the CRC microenvironment and oral delivery to advance the clinical translation of this strategy.

## Data Availability

Data will be made available on request.

## Abbreviations

5-FU: 5-Fluorouracil
ABPA1: *Aloe vera* Acetylated mannan
ABTS⁺: 2,2′-Azino-bis(3-ethylbenzothiazoline-6-sulfonic acid) radical cation
ACP: *Agrocybe cylindracea* Fucoglucogalactan
*AMPK*: AMP-activated protein kinase
APS: *Astragalus membranaceus* Polysaccharide
Ara: Arabinose
AS: *Angelica sinensis* Polysaccharide
ATP: Adenosine triphosphate
*Bad*: BCL2-associated agonist of cell death
*BAG4*: BCL2‑associated athanogene 4
*Bax*: BCL2-associated X protein
*Bcl**-**2*: B-cell lymphoma 2
*Bcl**-**xL*: B-cell lymphoma-extra large
*Bid*: BH3 interacting domain death agonist
*BIP*: Binding immunoglobulin protein
*BNIP3*: BCL2 interacting protein 3
*CHOP*: C/EBP homologous protein
*CpG*: Cytosine-phosphate-Guanine oligodeoxynucleotide
CPP: *Cyclocarya paliurus* Polysaccharide
CRC: Colorectal cancer
CS: Chitosan
*CTSD*: Cathepsin D
CTX: Cyclophosphamide
CVP-AP-I: A pectic polysaccharide isolated from *Chuanminshen violaceum*
DAO: Diamine oxidase
DCs: Dendritic cells
DES: Deep eutectic solvent
DHP: *Dendrobium huoshanense* Polysaccharide
DOP: A polysaccharide from *Dendrobium officinale*
DOX: Doxorubicin
DP: Dandelion root polysaccharide
DPPH: 2,2-Diphenyl-1-picrylhydrazyl
*Drp1*: Dynamin-related protein 1
DRP: Dandelion Root Polysaccharides
Epi: Epirubicin
EPS: *Cordyceps sinensis* Extracellular polysaccharides
ER: Endoplasmic reticulum
*E**RK*: Extracellular signal-regulated kinase
FFP@AgNPs: *Tussilago farfara* Polysaccharide silver nanoparticles
FU: Fucoidan
G0: Quiescent phase
G1: Gap 1 phase
GA-CDB: Gallic acid-chitosan oligosaccharide-dithiopropionate acid-berberine
GLP: *Ganoderma lucidum* Polysaccharide
H3K27ac: Histone H3 lysine 27 acetylation
HA: Hyaluronic acid
*HINT2*: Histidine triad nucleotide-binding protein 2
*HMGB1*: High mobility group box 1
ICD: Immunogenic cell death
IECs: Intestinal epithelial cells
IL: Interleukin
*IRE1α*: Inositol-requiring enzyme 1α
IRT: Irinotecan
*JAK2*: Janus kinase 2
LBP: *Lycium barbarum* Polysaccharide
*LC3**-**II*: Microtubule-associated protein 1 light chain 3-II
LMW‑AP‑FBG: Low-molecular-weight β-1,3-1,6-glucan derived from *Aureobasidium pullulans*
LNT: Lentinan
LNT-UA: Lentinan-ursolic acid
LP: Longan polysaccharide
MAMs: Mitochondria-associated membranes
Man: Mannose
*MCU*: Mitochondrial calcium uniporter
MDSCs: Myeloid-derived suppressor cells
MGN-3: Modified rice bran arabinoxylan
*miR**-**17**-**5p*: MicroRNA-17-5p
*MLCK*: Myosin light chain kinase
mPEG-PLA: Methoxy poly(ethylene glycol)-poly(lactic acid)
mPTP: Mitochondrial permeability transition pore
mtDNA: Mitochondrial DNA
*mTOR*: Mechanistic target of rapamycin
NAD⁺: Nicotinamide adenine dinucleotide
*ND1*: NADH dehydrogenase subunit 1
*NF**-**κB*: Nuclear factor kappa-light-chain-enhancer of activated B cells
NPs: Nanoparticles
*NR1D1*: Nuclear receptor subfamily 1 group D member 1
*Nrf2*: Nuclear factor erythroid 2‑related factor 2
OXA: Oxaliplatin
O₂•⁻: Superoxide anion radical
*p62*: Sequestosome-1
*Parkin*: PARK2 (E3 ubiquitin ligase)
*PARP*: Poly(ADP-ribose) polymerase
PEG: Polyethylene glycol
*PERK*: PKR-like ER kinase
PGPs-NE: Pomegranate polysaccharide nanoemulsion
*PINK1*: PTEN-induced putative kinase 1
PK/PD: Pharmacokinetic/pharmacodynamic
*PMI*: Phosphomannose isomerase
*PRRs*: Pattern recognition receptors
*RAGE*: Receptor for advanced glycation end products
RG-I: Rhamnogalacturonan I
ROS: Reactive oxygen species
SCP3: Sulfated *Cyclocarya paliurus* polysaccharide 3
SeNPs: Selenium nanoparticles
*SOD*: Superoxide dismutase
SOR: Sorafenib
SPF: Soy polysaccharide fiber
*STAT3*: Signal transducer and activator of transcription 3
SVP-A-1: A polysaccharide from *Sanghuangporus vaninii*
TCL: Tumor cell lysate
T*FAM*: Mitochondrial transcription factor A
*TFEB*: Transcription factor EB
Th1: T helper 1
Th2: T helper 2
*TLR*: Toll-like receptor
*TNF**-**α*: Tumor necrosis factor alpha
TPP: Triphenylphosphine
TPPP: Protoporphyrin-IX attached with Triphenylphosphonium
*TRAP1*: TNF receptor‑associated protein 1
*UGT8*: Uridine diphosphate‑glycosyltransferase 8
URP70-1: *Uncaria rhynchophylla* Polysaccharide
*USP14*: Ubiquitin‑specific protease 14
*ZNF746*: Zinc finger protein 746
